# Supplementary material for: Transbilayer Movement of Sphingomyelin Precedes Catastrophic Breakage of Enterobacteria-Containing Vacuoles
Source: Curr Biol. 2020 Aug 3;30(15):2974–2983.e6. doi: 10.1016/j.cub.2020.05.083 (PMC7416114; doi:10.1016/j.cub.2020.05.083)
Supplement: Document S1. Figures S1 and S2 and Table S1 [file mmc1.pdf]

Current Biology, Volume *30*

Supplemental Information

Transbilayer Movement of Sphingomyelin Precedes Catastrophic Breakage of Enterobacteria-Containing Vacuoles

Cara J. Ellison, Wanda Kukulski, Keith B. Boyle, Sean Munro, and Felix Randow

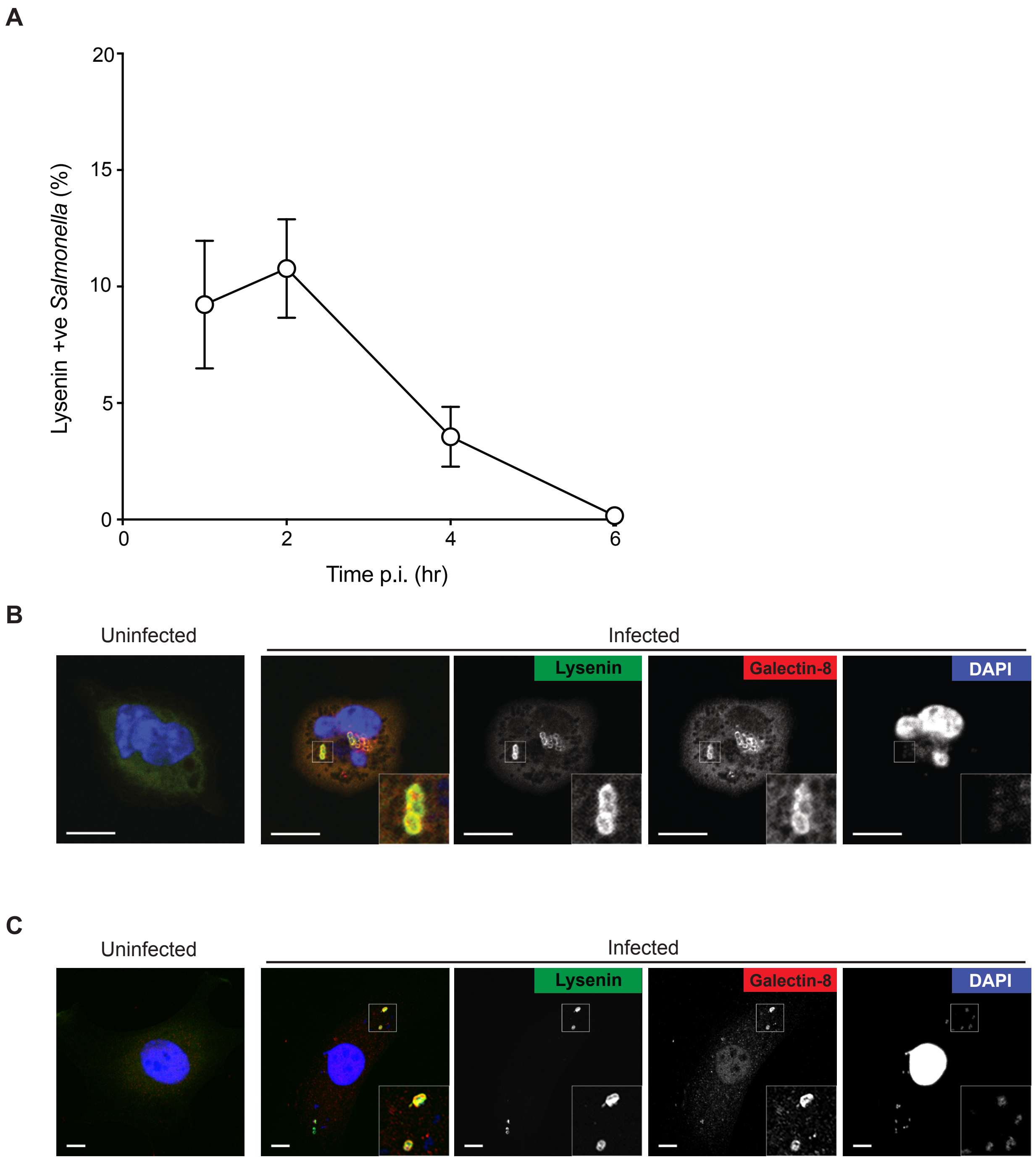

Figure S1: Lysenin recruitment to SCVs in HeLa, myeloid cells and fibroblasts, related to Figure 2

(A) Quantification of Lysenin^{W20A} recruitment to *S.* Typhimurium in HeLa cells at indicated time points p.i.. Mean ± SEM of triplicate coverslips from three independent experiments. Quantification by eye using widefield microscopy. n>100 bacteria counted per coverslip.

(B) Confocal micrographs of THP-1 cells expressing GFP-Lysenin^{W20A} infected with *S.* Typhimurium and stained for galectin-8 at 60 minutes p.i.. Scale bar, 10µm.

(C) Confocal micrographs of mouse embryonic fibroblast cells expressing GFP-Lysenin^{W20A} infected with *S.* Typhimurium and stained for galectin-8 at 60 minutes p.i.. Scale bar, 10µm.

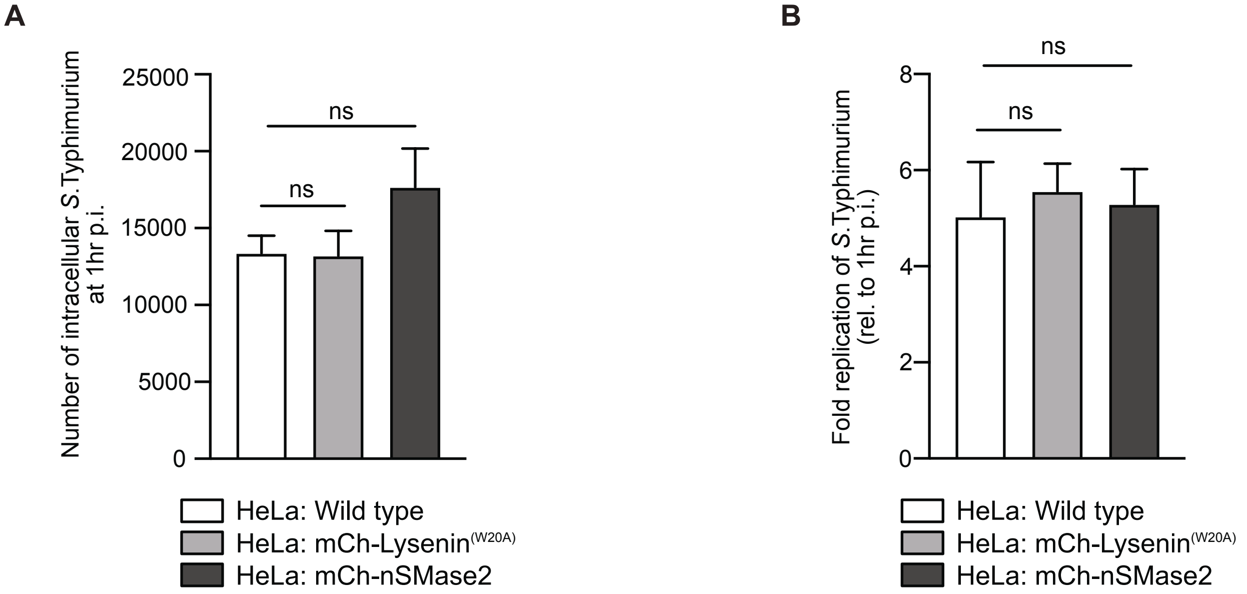

Figure S2: Ectopic expression of Lysenin or nSMase2 does not affect invasion by, or replication of, *S.* Typhimurium, related to Figure 2

(A) Quantification of intracellular *S*. Typhimurium in wild-type HeLa cells or HeLa cells expressing mCh-Lysenin^{W20A} or mCh-nSMase2 at 1 hour p.i.. Bacteria were counted in a colony forming unit assay. Mean ± SEM. One-way ANOVA with Dunnett's T3 multiple comparison test, ns = non-significant.

(B) Fold replication of *S*. Typhimurium in wild-type HeLa cells or HeLa cells expressing mCh-Lysenin^{W20A} or mCh-nSMase2 at 6hr p.i. relative to 1 hour p.i.. Bacteria were counted in a colony forming unit assay. Mean ± SD. One-way ANOVA with Dunnett's T3 multiple comparison test, ns = non-significant.

Oligonucleotides
Primer - Lysenin forward:<br>GGCCGGACATGTCCTCTGCCAAGGCCGCCGAGGGC
Primer - Lysenin reverse:<br>CCGGCCGCGGCCGCTCAGCCCACGACTTCCAGGAT
Primer - Lysenin CTD forward:<br>GGCCGGACATGTCCATCATCCTGGGAAAGACCGAG
Primer - nSMase2 forward:<br>GGCCGGACATGTCCGTTTTGTACACGACCCCCTTTCC
Primer - nSMase2 reverse:<br>CCGGCCGCGGCCGCCTATGCCTCCTCCTCCCCCGAAGA
Primer - Lysenin W20A mutation forward:<br>GATGTGGTGGCCGTG**GCG**AAAGAGGGCTATGTG
Primer - Lysenin W20A mutation reverse:<br>CACATAGCCCTCTTT**CGC**CACGGCCACCACATC
Primer - Lysenin K185A mutation forward:<br>ACCGTGGTGTCCAGA**GCG**AGCTGGCCTGCCGCC
Primer - Lysenin K185A mutation reverse:<br>GGCGGCAGGCCAGCT**CGC**TCTGGACACCACGGT
Primer - Lysenin CTD-GFP into pOPIN K at NcoI,HindIII sites - forward:<br>GGCCGGCCATGGCCATCATCCTGGGAAAGACC
Primer - Lysenin CTD-GFP into pOPIN K at NcoI,HindIII sites - reverse:<br>CCGGCCAAGCTTTCAATGATGATGATGATGATGGCCGGAGCCCTTGTACAGCTCGTCCAT
Primer - GFP into pET-M11 forward:<br>GGCCGGACATGTCCGTGAGCAAGGGCGAGGAGCTG
Primer - GFP into pET-M11 reverse:<br>CCGGCCGCGGCCGCTTACTTGTACAGCTCGTCCAT
Primer - GFP-Galectin 8 into pET-M11 forward:<br>GGCCGGACGCGTATGATGTTGTCCTTA
Primer - GFP-Galectin 8 into pET-M11 reverse:<br>CCGGCCGCGGCCGCCTACCAGCTCCTTACTTC
Primer - bSMase into pOPIN B forward:<br>*AAGTTCTGTTTCAGGGCCCG*ATGGAAAGCAAAAAAGATGACACCGAT
Primer - bSMase into pOPIN B reverse:<br>*ATGGTCTAGAAAGCTTTA*TTTGCTGTAGGCTTTAATC

Table S1: Oligonucleotides used in this study, related to STAR Methods.
