# Supplementary material for: Transbilayer Movement of Sphingomyelin Precedes Catastrophic Breakage of Enterobacteria-Containing Vacuoles
Source: Curr Biol. 2020 Aug 3;30(15):2974–2983.e6. doi: 10.1016/j.cub.2020.05.083 (PMC7416114; doi:10.1016/j.cub.2020.05.083)
Supplement: Document S2. Article plus Supplemental Information [file mmc8.pdf]

Report

Current Biology

Transbilayer Movement of Sphingomyelin Precedes Catastrophic Breakage of Enterobacteria-Containing Vacuoles

Graphical Abstract

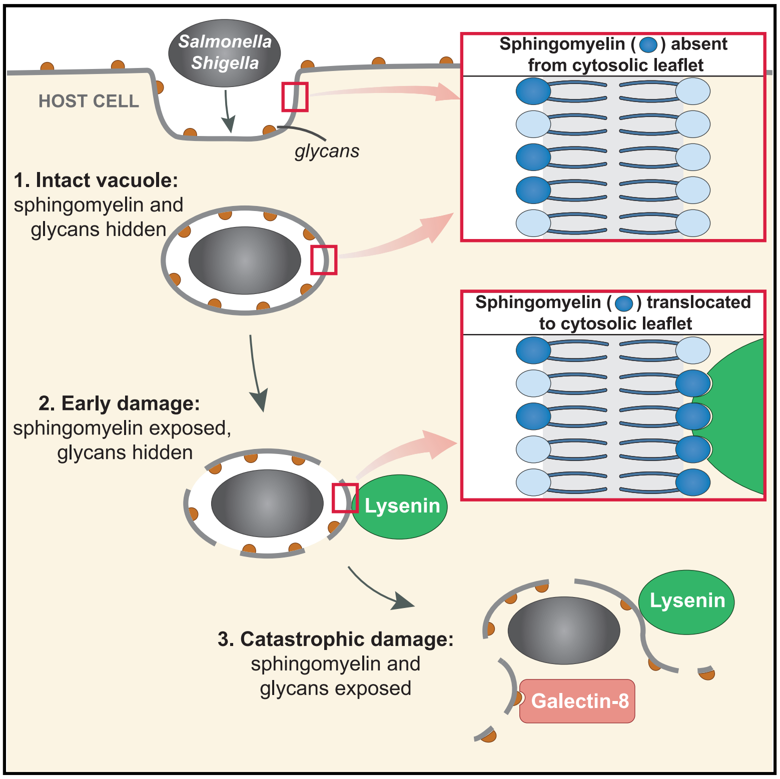

Highlights

- Lysenin serves as a reporter of sphingomyelin exposure in the mammalian cytosol
- Chemical-, toxin-, or pathogen-induced membrane damage exposes sphingomyelin
- Sphingomyelin exposure precedes catastrophic breakage of bacteria-containing vacuoles
- Cytosolic sphingomyelin is indicative of membrane stress and imminent pathogen entry

Authors

Cara J. Ellison, Wanda Kukulski, Keith B. Boyle, Sean Munro, Felix Randow

Correspondence

randow@mrc-lmb.cam.ac.uk

In Brief

Ellison et al. show that the pore-forming toxin Lysenin can be used as a reporter for detecting sphingomyelin in the cytosolic leaflet of cellular membranes. The breakout of Gram-negative bacteria from vacuoles into the host cytosol follows a precisely choreographed process in which sphingomyelin exposure precedes catastrophic membrane damage.

Ellison et al., 2020, Current Biology *30*, 2974–2983
August 3, 2020 © 2020 MRC Laboratory of Molecular Biology.
Published by Elsevier Inc.
https://doi.org/10.1016/j.cub.2020.05.083

CellPress

CellPress
OPEN ACCESS

Current Biology

Report

Transbilayer Movement of Sphingomyelin Precedes Catastrophic Breakage of Enterobacteria-Containing Vacuoles

Cara J. Ellison,[1,4] Wanda Kukulski,[2] Keith B. Boyle,[1] Sean Munro,[2] and Felix Randow[1,3,5,*]
[1]MRC Laboratory of Molecular Biology, Division of Protein and Nucleic Acid Chemistry, Francis Crick Avenue, Cambridge CB2 0QH, UK
[2]MRC Laboratory of Molecular Biology, Cell Biology Division, Francis Crick Avenue, Cambridge CB2 0QH, UK
[3]University of Cambridge, Department of Medicine, Addenbrooke's Hospital, Cambridge CB2 0QQ, UK
[4]Present address: Sir William Dunn School of Pathology, University of Oxford, Oxford OX1 3RE, UK
[5]Lead Contact
*Correspondence: randow@mrc-lmb.cam.ac.uk
https://doi.org/10.1016/j.cub.2020.05.083

SUMMARY

Pathogenic bacteria enter the cytosol of host cells through uptake into bacteria-containing vacuoles (BCVs) and subsequent rupture of the vacuolar membrane [1]. Bacterial invaders are sensed either directly, through cytosolic pattern-recognition receptors specific for bacterial ligands, or indirectly, through danger receptors that bind host molecules displayed in an abnormal context, for example, glycans on damaged BCVs [2–4]. In contrast to damage caused by *Listeria monocytogenes*, a Gram-positive bacterium, BCV rupture by Gram-negative pathogens such as *Shigella flexneri* or *Salmonella* Typhimurium remains incompletely understood [5, 6]. The latter may cause membrane damage directly, when inserting their Type Three Secretion needles into host membranes, or indirectly through translocated bacterial effector proteins [7–9]. Here, we report that sphingomyelin, an abundant lipid of the luminal leaflet of BCV membranes, and normally absent from the cytosol, becomes exposed to the cytosol as an early predictive marker of BCV rupture by Gram-negative bacteria. To monitor subcellular sphingomyelin distribution, we generated a live sphingomyelin reporter from Lysenin, a sphingomyelin-specific toxin from the earthworm *Eisenia fetida* [10, 11]. Using super resolution live imaging and correlative light and electron microscopy (CLEM), we discovered that BCV rupture proceeds through two distinct successive stages: first, sphingomyelin is gradually translocated into the cytosolic leaflet of the BCV, invariably followed by cytosolic exposure of glycans, which recruit galectin-8, indicating bacterial entry into the cytosol. Exposure of sphingomyelin on BCVs may therefore act as an early danger signal alerting the cell to imminent bacterial invasion.

RESULTS

Subcellular compartmentalization enables the generation of steep concentration gradients across compartment borders, which cells use to monitor compartment integrity [2]. The mammalian cytosol, for example, is devoid of complex glycans that, under homeostatic conditions, localize exclusively to the non-cytosolic leaflet of host membranes. Breakage of BCVs exposes these otherwise hidden glycans resulting in recruitment of galectins, a family of cytosolic lectins [12, 13]. Galectin-8 serves as a danger receptor and an "eat-me" signal detected by the autophagy cargo receptor NDP52, which directs selective autophagy against damaged BCVs and the bacteria contained therein [14–17]. Similar to glycans, certain lipids are also asymmetrically distributed in biological membranes [18]: sphingomyelin is enriched in the outer leaflet of the plasma membrane while phosphatidylserine and phosphatidylinositol are located primarily in the inner, cytoplasmic leaflet. The translocation of phosphatidylserine to the outer leaflet during apoptosis marks dying cells and leads to their timely removal [19]. Since the asymmetric distribution of host molecules across biological membranes contains information about the integrity and functionality of organelles and cells, further investigations into dynamic changes in asymmetrically distributed host components seem advisable. We speculated that during the cytosolic entry of bacteria, sphingomyelin becomes cytosolically exposed on damaged BCVs, analogous to glycans, where it could provide a novel danger signal indicating membrane stress.

Lysenin Detects Exposure of Sphingomyelin to the Cytosol

To visualize whether sphingomyelin becomes exposed to the cytosol upon endomembrane damage, we developed a fluorescent sphingomyelin reporter based on Lysenin, a sphingomyelin-binding member of the aerolysin family of β-pore-forming toxins from the earthworm *Eisenia fetida* [10, 11]. Lysenin binds sphingomyelin specifically and with high affinity through its C terminus, before oligomerizing via its N terminus into a nonameric pore [11, 20–22]. To enable expression of the otherwise toxic protein, we deployed either the isolated C-terminal domain

 © 2020 MRC Laboratory of Molecular Biology. Published by Elsevier Inc.
This is an open access article under the CC BY license (http://creativecommons.org/licenses/by/4.0/).

CellPress
OPEN ACCESS

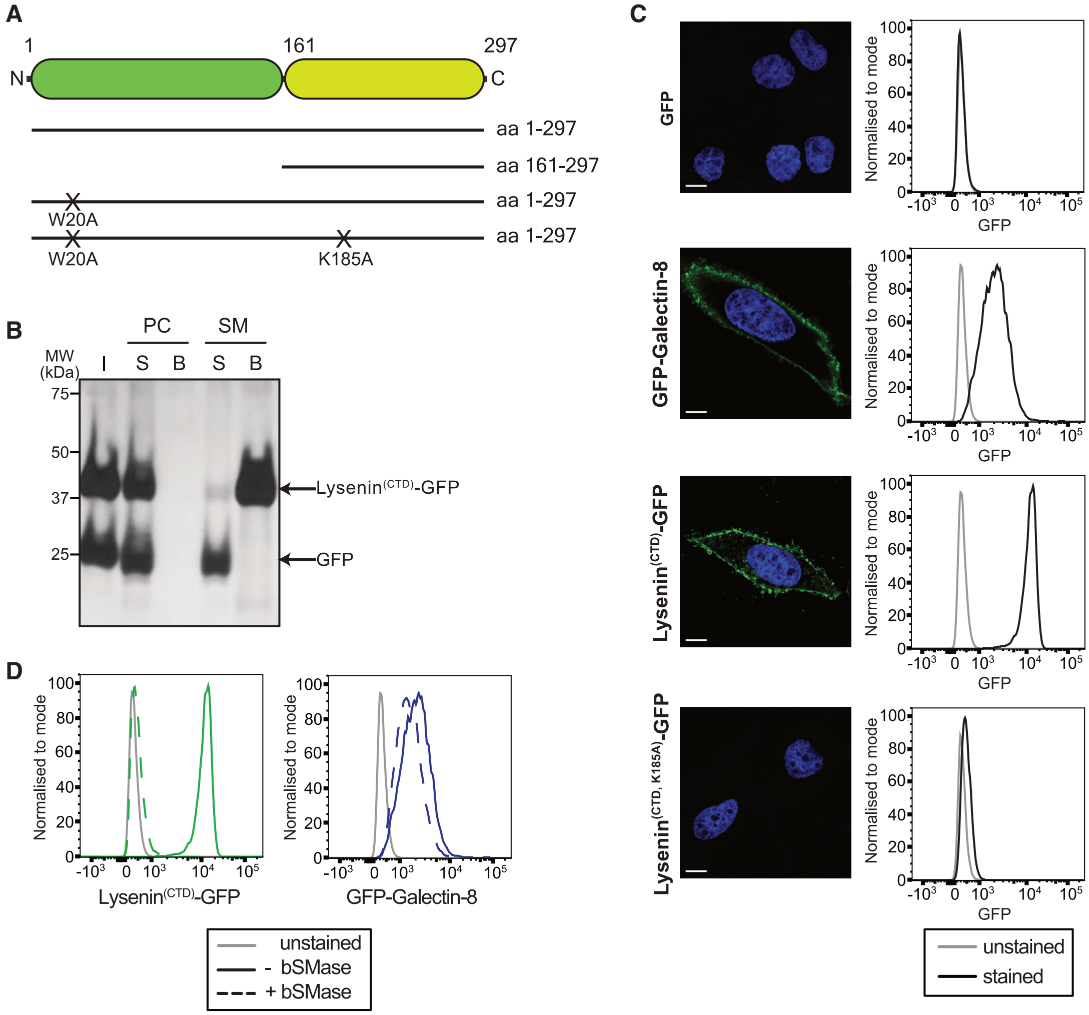

Figure 1. Lysenin Specifically Binds Sphingomyelin

(A) Constructs of Lysenin used in this study. Green, N-terminal domain; yellow, C-terminal domain.

(B) Liposome flotation assay. Liposomes containing PC:Cholesterol (labeled PC) or Sphingomyelin:PC:Cholesterol (labeled SM) were mixed with recombinant Lysenin^{CTD}-GFP purified from *E. coli*. After incubation, liposomes were floated and harvested. Proteins extracted from supernatant or liposomes were visualized by silver stain. I, input; S, supernatant; B, bound.

(C) Cell-surface binding assay. Non-permeabilized HeLa cells were incubated with recombinant GFP, GFP-Galectin-8, Lysenin^{CTD}-GFP, or Lysenin^{CTD,K185A}-GFP, washed, and fixed. Binding was assessed by confocal microscopy and flow cytometry. Scale bar, 10 μm. Gray line, unstained; black line, stained with corresponding recombinant protein.

(D) Effect of bSMase on Lysenin binding. Flow cytometry of untreated cells or cells pretreated with recombinant bacterial SMase followed by incubation with recombinant GFP-Galectin-8 or Lysenin^{CTD}-GFP.

(CTD) (residues 161–297, Lysenin^{CTD}) or a full-length oligomerization-deficient mutant (Lysenin W20A), both known to retain sphingomyelin binding activity [23–25] (Figure 1A).

We verified the specificity of our Lysenin construct by three means: (1) recombinant Lysenin^{CTD}-GFP bound sphingomyelin-containing liposomes but not those devoid of sphingomyelin (Figure 1B), (2) binding of recombinant Lysenin^{CTD}-GFP to the extracellular surface of HeLa cells was abrogated by pretreatment with bacterial sphingomyelinase (bSMase), whereas binding of GFP-galectin-8 was not (Figures 1C and 1D), and (3) Lysenin^{CTD,K185A}-GFP, a mutant based on the structure of Lysenin bound to the phosphocholine head group of sphingomyelin [20], did not bind to the extracellular surface of cells (Figure 1C). Taken together, we conclude that Lysenin specifically binds sphingomyelin and that no other Lysenin ligand is present on the extracellular face of the plasma membrane.

Salmonella-Containing Vacuoles Recruit Lysenin in a Sphingomyelin-Dependent Manner

To enable the detection of sphingomyelin in the cytosol of cells, we generated reporter lines stably expressing GFP-Lysenin^{CTD} or GFP-Lysenin^{W20A} (Figure 2A). Both constructs were diffusely distributed in control cells, although in some cells small punctae were apparent, which may be due to either a mild tendency of

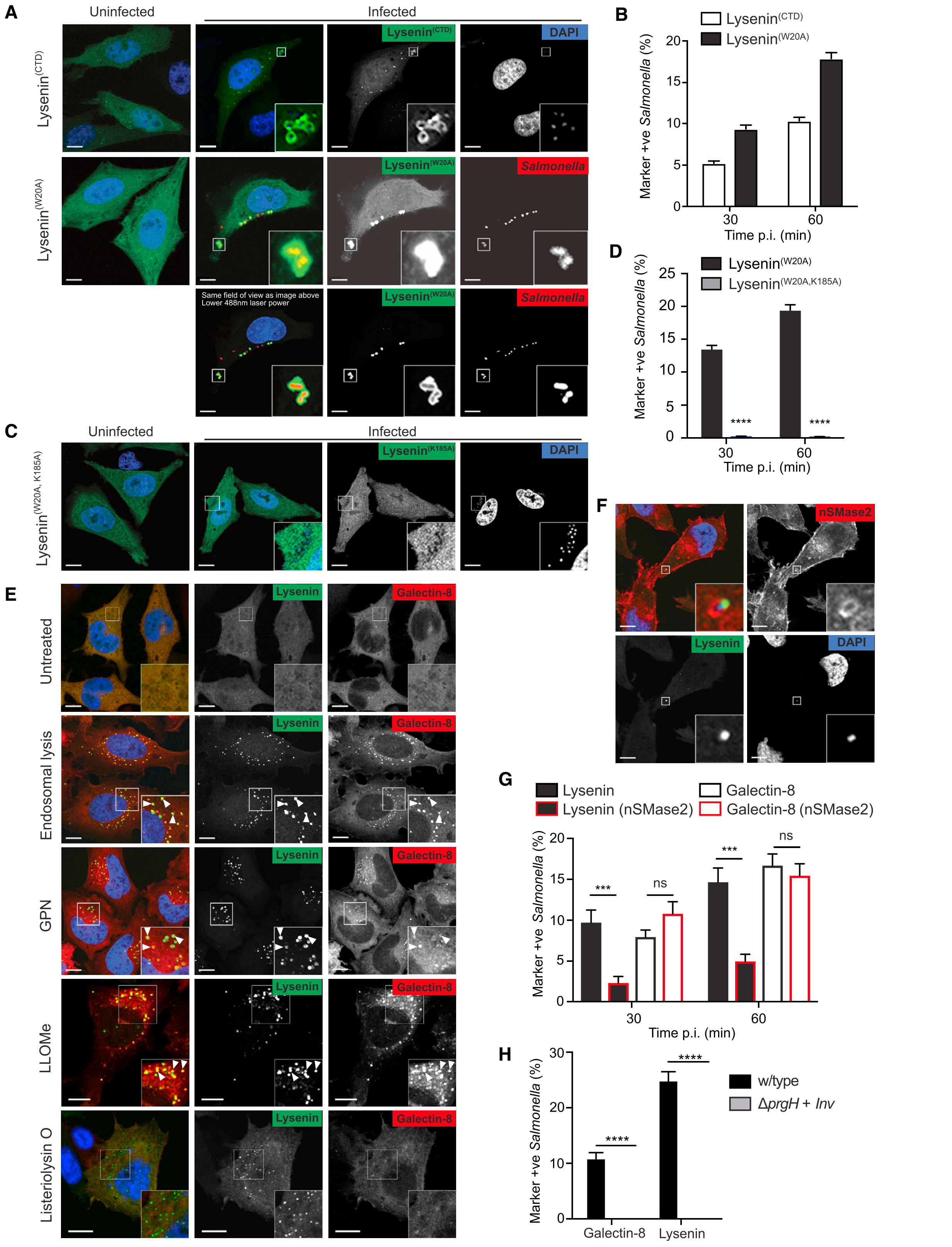

(legend on next page)

CellPress
OPEN ACCESS

Lysenin to aggregate upon overexpression or sphingomyelin exposure upon spontaneous membrane damage.

We next investigated whether sphingomyelin becomes cytosolically exposed during membrane damage caused by invasive bacteria. *Salmonella enterica* serovar Typhimurium (*S.* Typhimurium), a Gram-negative enterobacterium, deploys a Type Three Secretion System (T3SS) to invade epithelial cells, where it establishes a membrane-surrounded compartment, the *Salmonella*-containing vacuole (SCV). T3SS-dependent damage to the SCV membrane results in recruitment of galectin-8 to cytosolically exposed glycans and provides cytosolic access for 10%–20% of the invading bacteria [14, 26, 27]. In epithelial cells infected with *S.* Typhimurium, we observed strong recruitment of GFP-Lysenin to a subset of bacteria as visualized by both live-cell imaging (Video S1) and analysis of fixed samples (Figure 2A). Lysenin recruitment peaked at 1–2 h post-infection (p.i.) and became negligible at 6 h p.i. (Figure S1A). At 60 min p.i., GFP-Lysenin^{CTD} and GFP-Lysenin^{W20A} labeled 10%–20% of SCVs (Figure 2B). The greater percentage labeling by GFP-Lysenin^{W20A} suggests greater sensitivity, making it the preferred Lysenin construct for our further investigations. In contrast, GFP-Lysenin^{W20A,K185A} was not recruited to SCVs (Figures 2C and 2D), consistent with the inability of Lysenin^{CTD,K185A} to bind sphingomyelin (Figure 1C).

To test whether damage to endomembranes under sterile conditions also recruits Lysenin, we subjected cells co-expressing GFP-Lysenin^{W20A} and the membrane damage marker mCherry-galectin-8 to a variety of membrane damaging conditions. Osmotic shock treatment, known to injure endosomes, or exposure to the lysosome-damaging drugs glycyl-L-phenylalanine 2-naphthylamide (GPN) or LLOMe resulted in accumulation of Lysenin^{W20A} in punctae that co-localized with galectin-8 (Figure 2E). In contrast, treatment with listeriolysin O, a hemolysin from *L. monocytogenes*, caused the formation of Lysenin^{W20A} punctae negative for galectin-8, indicative of less severe membrane damage than inflicted by osmotic shock, GPN, or LLOMe. We conclude that damage to endosomal or lysosomal membranes causes exposure of sphingomyelin to the cytosol that can be visualized through Lysenin accumulation.

Infection of myeloid THP1 cells or fibroblasts with *S.* Typhimurium also caused recruitment of GFP-Lysenin to SCVs, indicating that sphingomyelin exposure on SCVs occurs in multiple cell types (Figures S1B and S1C).

Neutral sphingomyelinase 2 (nSMase2) is a transmembrane sphingomyelin hydrolyzing enzyme whose active site is located in the cytosol [28, 29]. Overexpression of nSMase2 significantly reduced Lysenin recruitment to SCVs without affecting galectin-8 (Figures 2F and 2G), the invasiveness of *S.* Typhimurium or its intracellular proliferation **(**Figure S2**)**. Overexpression of Lysenin did not affect the invasiveness or proliferative capacity of *S.* Typhimurium either **(**Figure S2**)**. We conclude that sphingomyelin becomes cytosolically exposed on damaged SCVs, where it is specifically detected by our Lysenin-based sphingomyelin reporter.

Sphingomyelin Exposure on SCVs Is T3SS Dependent

We next investigated the cause of sphingomyelin exposure on SCVs. Bacterial secretion systems, including the SPI1 T3SS of *S.* Typhimurium, can damage host membranes [8, 9, 30]. Notably, neither Lysenin nor galectin-8 were recruited to *S.* Typhimurium Δ*prgH* + *Inv*, a strain that invades epithelial cells by means of the *Yersinia* invasin gene, *Inv*, but remains confined to the SCV because of a non-functional SPI1 needle [31, 30, 32] (Figure 2H). We conclude that SPI1-mediated damage of SCVs results in cytosolic exposure of sphingomyelin.

Sphingomyelin Is Exposed on Vacuoles Containing Gram-Negative or Gram-Positive Bacteria

We next investigated whether exposure of sphingomyelin occurred during cytosolic invasion by bacteria other than *S.* Typhimurium. GFP-Lysenin^{W20A} was also recruited to *Shigella flexneri*, *Listeria monocytogenes*, and *Streptococcus pyogenes*, i.e., both Gram-negative and Gram-positive species (Figures 3A and 3B). In contrast, infection with Enteropathogenic *E. coli* (EPEC) did not cause Lysenin accumulation in the bacterial

Figure 2. Lysenin Is Recruited to Bacteria-Containing Vacuoles in a Sphingomyelin-Dependent Manner

(A) Confocal micrographs of HeLa cells expressing GFP-Lysenin^{CTD} or GFP-Lysenin^{W20A} either uninfected or infected with mCherry-expressing *S.* Typhimurium 12023 and analyzed at 30 min post-infection (p.i.). Two micrographs of the same field of view are presented for HeLa cells expressing GFP-Lysenin^{W20A} and infected with *S.* Typhimurium. The upper image was acquired with identical settings to the uninfected control; the lower image was acquired with a reduced 488 nm laser power. Scale bar, 10 μm.

(B) Percentage of *S.* Typhimurium positive for Lysenin^{CTD} or Lysenin^{W20A} at 30 and 60 min p.i. Mean ± SEM of triplicate wells from three independent repeats. Automated image acquisition, automated quantification. n > 6,000 bacteria counted per well.

(C) Confocal micrographs of HeLa cells expressing GFP-Lysenin^{W20A,K185A} either uninfected or infected with mCherry-expressing *S.* Typhimurium and analyzed at 30 min post-infection (p.i.). Scale bar, 10 μm.

(D) Percentage of *S.* Typhimurium positive for Lysenin^{W20A} or Lysenin^{W20A,K185A} at 30 and 60 min p.i. Mean ± SEM of triplicate wells from three independent repeats. Automated image acquisition, automated quantification. n > 6,000 bacteria counted per well. ****$p < 0.0001$, Student's t test.

(E) Confocal micrographs of HeLa cells expressing GFP-Lysenin^{W20A} and mCherry-galectin-8 following treatment with different sterile damage-inducing reagents. White arrows indicate examples of Lysenin and galectin-8 co-localization. Scale bar, 10 μm

(F) Confocal micrographs of HeLa cells expressing GFP-Lysenin^{W20A} and mCherry-nSMase2 infected with *S.* Typhimurium and analyzed at 60 min p.i. Scale bar, 10 μm.

(G) Percentage of *S.* Typhimurium positive for Lysenin^{W20A} or galectin-8 in the presence or absence of ectopically expressed nSMase2. Mean ± SEM of triplicate wells from three independent repeats. Automated image acquisition, manual quantification. n > 700 bacteria counted per well. Ns, non-significant; ***$p < 0.001$, Student's t test.

(H) Percentage of S. Typhimurium wild-type (w/type) or Δ*prgH* + *Inv* positive for Lysenin^{W20A} or galectin-8. Mean ± SEM of triplicate coverslips from three independent repeats. Quantification by eye using wide-field microscopy. n > 200 (w/type), n > 45 (Δ*prgH* + *Inv*) bacteria counted per coverslip. ****$p < 0.0001$, Student's t test.

See also Video S1 and Figures S1 and S2.

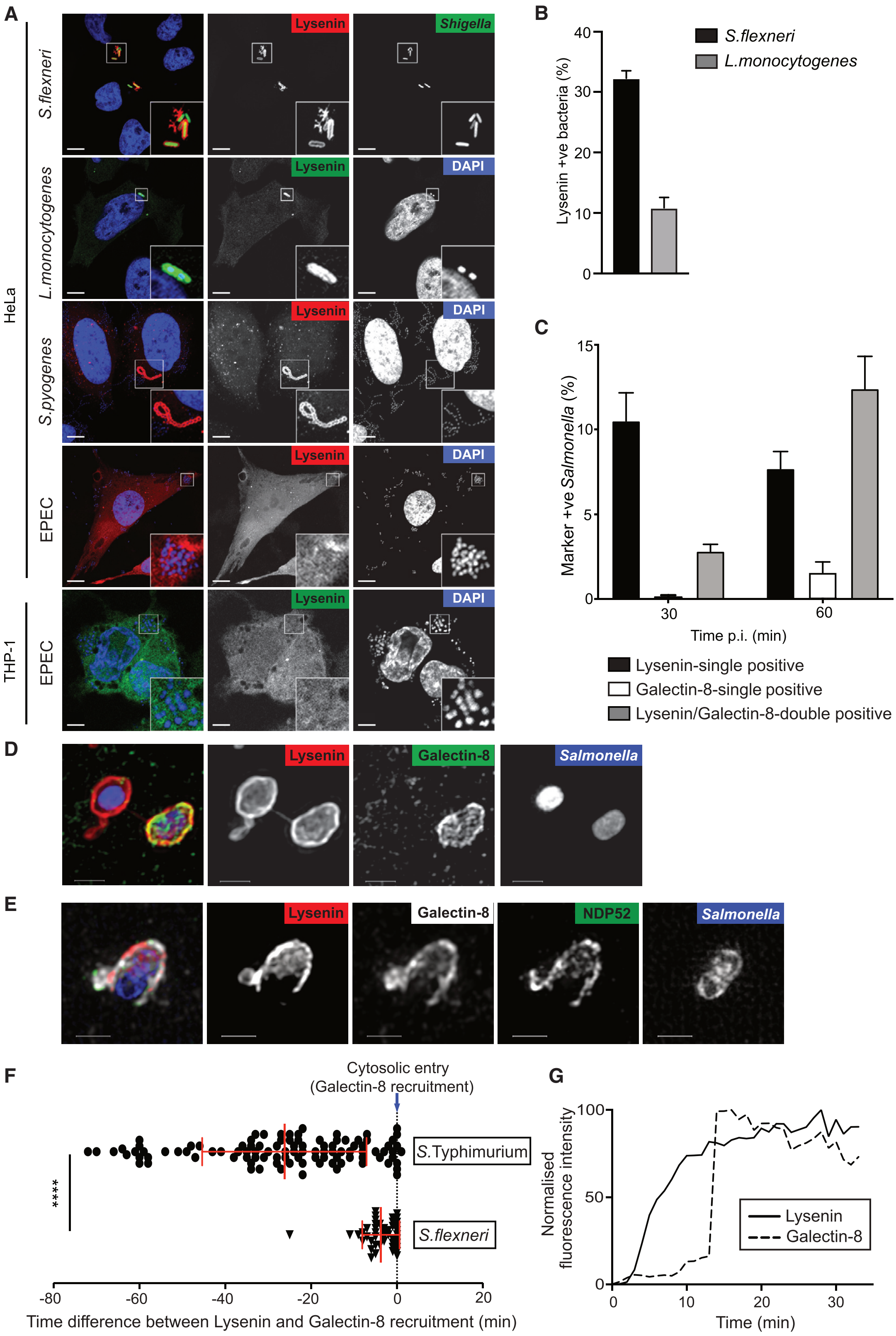

(legend on next page)

CellPress
OPEN ACCESS

vicinity irrespective of whether bacteria interacted with the plasma membrane of epithelial cells or became phagocytosed by myeloid cells (Figure 3A) revealing that not all pathogenic bacteria cause the translocation of sphingomyelin even if they encode a functional T3SS. Live-cell imaging revealed that in the case of *Shigella flexneri*, the Lysenin "coat" was ultimately shed by the bacterium, consistent with Lysenin detecting sphingomyelin on the damaged vacuolar membrane (Video S2). We therefore conclude that cytosolic entry of both Gram-positive and Gram-negative bacteria causes cytosolic sphingomyelin exposure on damaged endomembranes.

Sphingomyelin Is Exposed on Damaged SCVs before Glycans

To investigate the process by which Gram-negative bacteria rupture their vacuole en route to the cytosol, we studied Lysenin recruitment to SCVs in the context of galectin-8 as a marker of membrane rupture. At 30 min p.i., the majority of labeled SCVs were Lysenin-single positive, while at 60 min p.i. most labeled SCVs had become Lysenin/galectin-8-double positive, revealing that Lysenin and galectin-8 target the same SCVs but Lysenin recruitment occurs prior to galectin-8 (Figures 3C and 3D). Lysenin/galectin-8-double positive SCVs also associated with the autophagy cargo receptor NDP52 indicating that these SCVs are targeted for autophagy (Figure 3E).

To further investigate the kinetics of endomembrane damage we followed individual bacteria by live-cell microscopy. Lysenin was recruited to SCVs prior to galectin-8, with an average time differential of 28 ± 19.2 min (Figure 3F; Video S3), indicating that T3SS-mediated damage to SCVs follows a precisely choreographed pathway in which the cytosolic exposure of sphingomyelin invariably precedes the exposure of glycans. Lysenin is therefore a marker of early SCV damage that is predictive of subsequent glycan exposure. Tracking revealed that Lysenin accumulated on SCVs in a gradual manner over several minutes, in contrast to galectin-8, which appeared abruptly (Figure 3G), suggesting a gradual transfer of sphingomyelin from the luminal to the cytosolic leaflet of SCV membranes, followed by the abrupt exposure of glycans.

To test whether the sequential recruitment of Lysenin and galectin-8 also occurs during the entry of other Gram-negative bacteria into the cytosol, we monitored the rupture of *S. flexneri*-containing vacuoles (SfCVs). Recruitment of Lysenin preceded galectin-8 but with a much shorter time differential (average 4 ± 4.7 min) than for *S.* Typhimurium (Figure 3F), indicating a faster entry process for the cytosol-adapted *S. flexneri*. Taken together, we conclude that cytosolic sphingomyelin exposure is an early and predictive feature of BCVs liable of releasing their content into the host cytosol.

Differential Exposure of Sphingomyelin and Glycans Define Two Stages of BCV Rupture

The slow and progressive accumulation of sphingomyelin on SCVs destined for glycan exposure prompted us to investigate whether the membranes of Lysenin-single positive SCVs are still intact and whether the subsequent abrupt appearance of galectin-8 marks a catastrophic breakdown of membrane integrity. In live-cell super resolution microscopy, Lysenin appeared initially as a seemingly complete ring, consistent with Lysenin recruitment to the cytosolic leaflet of the SCV before any marked membrane damage (Figure 4A; Video S4), a conclusion further supported by the ability of cytosolically active nSMase2 to antagonize Lysenin recruitment (Figures 2F and 2G). The ultimate loss of homogeneity in the Lysenin ring coincided with recruitment of galectin-8, which is consistent with a break in the SCV membrane resulting in glycan exposure (Figure 4A). We obtained similar data for *S. flexneri*, where Lysenin was recruited to a seemingly complete SfCV prior to galectin-8 recruitment that coincided with disruption of the homogeneous Lysenin distribution (Figure 4B).

To visualize the SCV membrane directly, we used correlative fluorescence microscopy and electron tomography [33] on SCVs that were either single positive for Lysenin or double positive for Lysenin and galectin-8. In fluorescence images of resin sections, the Lysenin-single positive signal appeared continuous around the SCV, consistent with super resolution imaging. Electron tomograms (n = 12) of these signals nevertheless revealed the existence of membrane gaps of 100–200 nm diameter in 2 of the 12 SCVs (Figure 4C; Video S5). Since we estimated that the membrane contained in each of these two tomograms comprises only approximately 11% of the total SCV surface, it is possible that the remaining 10 SCVs also contain gaps, despite their membrane appearing intact within the tomograms. In contrast, electron tomograms of Lysenin/galectin-8-double positive SCVs, which in fluorescence microscopy displayed a

Figure 3. Sphingomyelin Is Exposed on Vacuoles Containing Gram-Negative or Gram-Positive Bacteria and Exposure Occurs before Glycans

(A) Confocal micrographs of HeLa cells expressing Lysenin^{W20A} infected with *Shigella flexneri* M90T, *Listeria monocytogenes* EGD (BUG 600), *Streptococcus pyogenes* H293, or Enteropathogenic *E. coli* E2348/69 (EPEC). Confocal micrograph of THP-1 cells expressing Lysenin^{W20A} infected with EPEC. HeLa scale bar, 10 μm; THP-1 scale bar, 5 μm.

(B) Quantification of Lysenin recruitment to *S. flexneri* and *L. monocytogenes*. Mean ± SEM of triplicate coverslips from three independent repeats. Quantification by eye using wide-field microscopy. n > 100 bacteria counted per coverslip.

(C) Percentage of *S.* Typhimurium positive for Lysenin^{W20A} and/or galectin-8 at 30 and 60 min p.i. Mean ± SEM of triplicate wells from three independent repeats. Automated image acquisition, manual quantification. n > 700 bacteria counted per well.

(D) Structured illumination micrographs of HeLa cells expressing mCherry-Lysenin^{W20A} and YFP-galectin-8 infected with blue fluorescent protein (BFP)-expressing *S.* Typhimurium and fixed at 60 min p.i.. Scale bar, 1 μm.

(E) Structured illumination micrographs of HeLa cells expressing mCherry-Lysenin^{W20A} infected with BFP-expressing *S.* Typhimurium and stained for galectin-8 and NDP52 at 60 min p.i.. Scale bar, 1 μm.

(F) Quantification of the time interval between Lysenin and galectin-8 recruitment to *S.* Typhimurium or *S. flexneri* visualized by live-cell imaging. n > 107 *S.* Typhimurium and n > 45 *S. flexneri* events were analyzed. Mean ± SD indicated. ****p < 0.0001, Student's t test.

(G) Tracking of a *Salmonella*-containing vacuole visualized by live-cell imaging. Graph indicates mean 488 nm (green) and 561 nm (red) fluorescence intensity changes around the bottom bacterium in Video S3. Time indicated is from the start of the track rather than bacterial entry into the cell. Graph shown is a representative example.

See also Video S3.

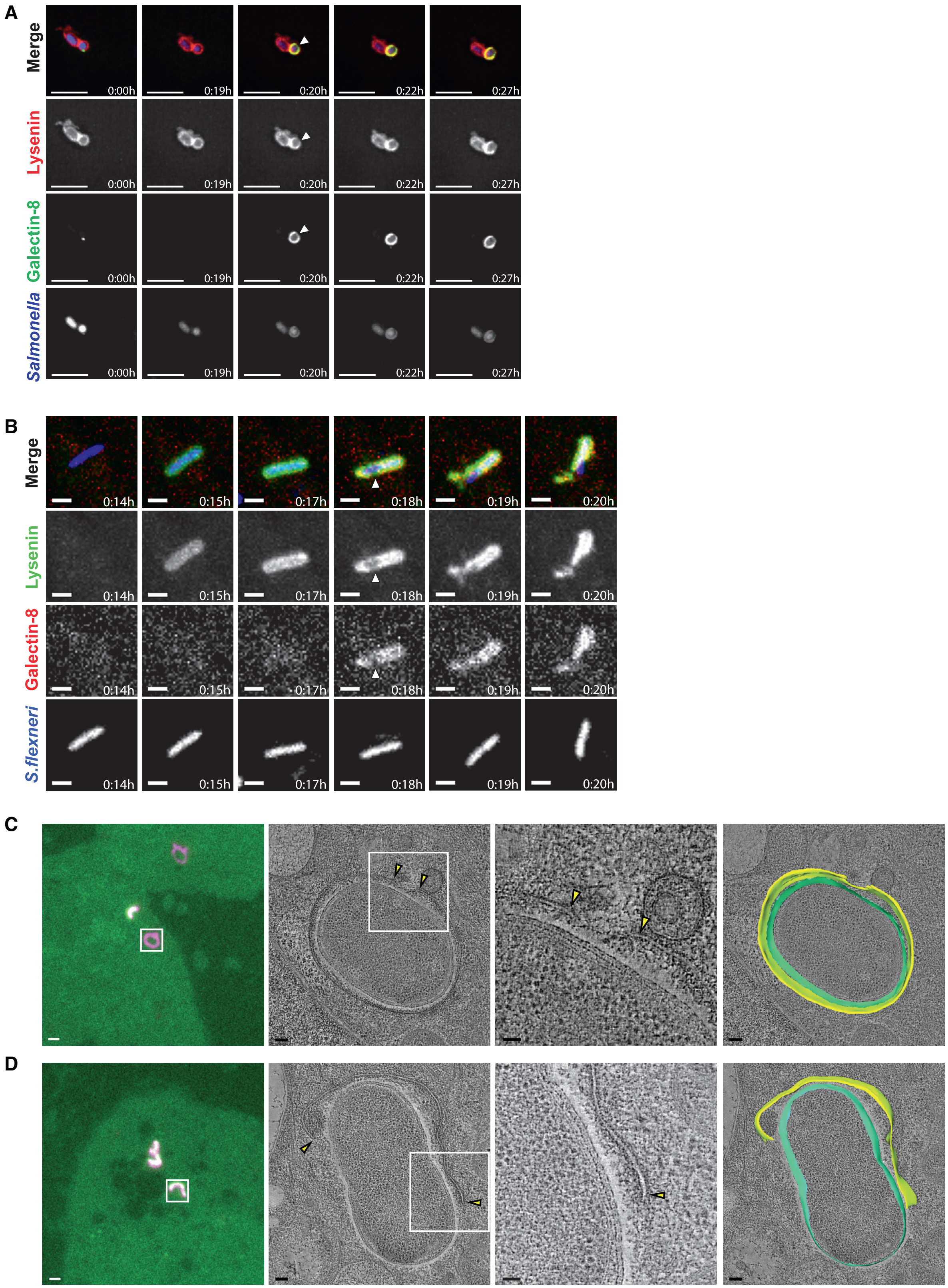

Figure 4. Sphingomyelin Is Exposed on the Cytosolic Leaflet of BCVs before Cytosolic Entry of the Bacterium

(A) Selected frames from super resolution live-cell imaging of HeLa cells expressing mCherry-Lysenin^{W20A} and YFP-galectin-8 infected with BFP-expressing *S.* Typhimurium shown in Video S4. White arrow indicates appearance of a break in the SCV membrane. Scale bar, 5 μm.

(legend continued on next page)

CellPress
OPEN ACCESS

complete overlap of the two signals and a clear signal gap (n = 5), revealed much larger membrane ruptures with the vacuolar membrane no longer enclosing the bacterium (Figure 4D; Video S6).

We therefore conclude that, for Gram-negative bacteria, the rupture of pathogen-containing vacuoles proceeds through two stages. The initial T3SS-dependent disruption of the SCV membrane permits the gradual exposure of sphingomyelin on the cytosolic face of the SCV. Sphingomyelin release may result from, or be causative of, the formation of localized ruptures of up to 200 nm. Importantly, these initial ruptures do not permit the interaction between luminal glycans and galectin-8, which only occurs on SCVs with large ruptures, indicative of a pronounced loss of SCV membrane integrity.

DISCUSSION

We have developed Lysenin, a sphingomyelin-specific toxin from earthworms, into a sensitive and specific reporter for the visualization of sphingomyelin in the cytosolic leaflet of cellular membranes. Using this new Lysenin reporter, we reveal that the transition of Gram-negative bacteria from their vacuole into the host cytosol follows a precisely choreographed multi-step process, in which cytosolic sphingomyelin exposure is invariably followed by catastrophic membrane damage, glycan exposure, and cytosolic entry of the bacterium.

Compared to the cytosol-adapted Gram-positive bacterium *Listeria monocytogenes*, escape of Gram-negative bacteria from their vacuole remains poorly understood. *Listeria* deploys the pore-forming toxin listeriolysin O and two phospholipases to destroy its vacuole, while Gram-negative bacteria cause membrane damage through their T3SSs [5, 8]. Secreted effector proteins are not essential for cytosolic entry of Gram-negative bacteria, at least not in *S. flexneri* where the secretion apparatus itself, and specifically the translocon proteins IpaB and IpaC, contributes to vacuolar rupture [8]. Pore formation through translocon proteins, akin to pore formation by listeriolysin O, may therefore be required to trigger vacuole lysis. However, translocon pores per se may not be sufficient to cause vacuolar lysis, as the T3SSs of *Shigella*, *Salmonella*, and *Yersinia*, despite all forming pores in host membranes, differ widely in their membranolytic potential. Effector proteins that antagonize cytosolic entry, such as SifA and SopF in *S.* Typhimurium, may further complicate the situation [27, 34]. Our discovery of sphingomyelin exposure as an early marker of vacuolar damage, invariably preceding the exposure of glycans and catastrophic breakdown of membrane integrity, sheds new light on the entry process of Gram-negative bacteria. The early and slow recruitment of Lysenin is indicative of the gradual appearance of sphingomyelin on the cytosolic face of the vacuole. How does sphingomyelin gradually transfer from the luminal to the cytosolic leaflet of the vacuole? The long half-life of sphingolipids and phospholipids for spontaneous leaflet transfer necessitates an assisted process, for example, through the action of a dedicated scramblase or as a result of the translocon pore being formed, which causes bending of the membrane as recently shown for the *Salmonella* SPI1 needle [35]. Assisted transfer of sphingomyelin from the luminal to the cytosolic leaflet may result in a net flow of lipids, creating an imbalance in the lateral tension of the two leaflets and ultimately destabilizing the membrane. Alternatively, sphingomyelin exposure may recruit cellular proteins that induce rupture. In either scenario, sphingomyelin transfer between leaflets would critically contribute to catastrophic vacuolar damage and thus entry of Gram-negative bacteria into the host cytosol. It is tempting to speculate that the membrane gaps visualized by electron tomography in Lysenin-positive, galectin-8-negative vacuoles are the result of sphingomyelin leaflet transfer, although we cannot exclude the possibility that said membrane gaps are themselves sites of lipid transfer between membrane leaflets.

Our experiments revealed the existence of two classes of membrane ruptures in BCVs: an early breakage in Lysenin-positive, galectin-8-negative membranes characterized by substantial, but still relatively small, gaps of up to 200 nm and subsequent, much larger breakages in Lysenin/galectin-8 double-positive membranes. The lack of galectin-8 recruitment to the former indicates that, despite the opening of substantial membrane gaps, a diffusion barrier still exists that prevents the cytosolic exposure of glycans as well as the entry of galectin-8 into the vacuolar lumen. Perhaps large protein complexes of annexins and other membrane repair proteins are formed at these rupture sites that stabilize the membrane edges, limit the size of the gaps, and provide a diffusion barrier [36]. However, since on BCVs sphingomyelin translocation invariably precedes glycan exposure, i.e. catastrophic rupture, repair of membrane gaps in Lysenin-positive membranes seems inefficient against bacterial entry. In contrast, repair of Lysenin-positive membranes may play a more important role in toxin-mediated damage as listeriolysin O-induced Lysenin punctae did not accumulate galectin-8.

The recruitment of galectin-8 to cytosolically exposed glycans provides a danger signal alerting cells to catastrophic endomembrane damage, often a telltale sign of pathogen invasion into the cytosol. Galectin-8 accumulation on such damaged membranes induces a localized inhibition of mTOR activity [37]

(B) Selected frames from live-cell imaging of HeLa cells expressing mCherry-Lysenin^{W20A} and CFP-galectin-8 infected with GFP-expressing *S. flexneri* shown in Video S2. Video and corresponding stills were false colored to aid visualization: green, Lysenin^{W20A}; red, galectin-8; blue, *S. flexneri.* White arrow indicates appearance of a break in the SfCV membrane. Scale bar, 2 μm.

(C and D) Correlative fluorescence and electron microscopy of HeLa cells expressing mCherry-Lysenin^{W20A} and YFP-galectin-8 infected with BFP-expressing *S.* Typhimurium 30–40 min post-infection. Left panel: fluorescence images of ~300 nm sections through resin-embedded cells, merge of red (mCherry signal) and green (YFP signal) channel. White square corresponds to area shown in second panel, imaged by electron tomography. Second panel: virtual slice through electron tomogram, showing SCV identified by fluorescence microscopy. White square corresponds to the area magnified in the third panel. Yellow arrows indicate ruptures of the SCV membrane. Right panel shows 3D segmentation models of the SCV membrane (yellow) and the surface of the bacterium (green). The background is a different virtual slice of the tomogram shown in the second panel. Scale bar, 1 μm (left panel), 100 nm (second and right panel), and 50 nm (third panel).

See also Videos S2, S4, S5, and S6.

and the induction of selective autophagy against membrane remnants and vacuolar cargo, e.g., pathogens [14, 16]. It remains to be explored whether sphingomyelin exposure to the cytosol also provides a danger signal. Indeed, abnormal cytosolic exposure of lipids such as cardiolipin on outer mitochondrial membranes signals mitochondrial damage and induces mitophagy [38]. Further investigations are needed to determine whether cytosolically exposed sphingomyelin signals through recruitment of mammalian sphingomyelin-binding proteins or whether it is converted into other bioactive sphingolipids such as ceramide and sphingosine.

STAR★METHODS

Detailed methods are provided in the online version of this paper and include the following:

- KEY RESOURCES TABLE
- RESOURCE AVAILABILITY
  - Lead Contact
  - Materials Availability
  - Data and Code Availability
- EXPERIMENTAL MODEL AND SUBJECT DETAILS
  - Cell lines
  - Bacteria
- METHOD DETAILS
  - Plasmids
  - Sterile damage assays
  - Bacterial infections
  - Enumeration of intracellular *S.* Typhimurium (colony forming unit assay)
  - Antibody staining
  - Microscopy
  - Protein expression and purification
  - Surface binding assays
  - Liposome flotation assays
  - Correlative Fluorescence Microscopy and Electron Tomography
- QUANTIFICATION AND STATISTICAL ANALYSIS
  - Quantification of bacteria
  - Enumeration of intracellular *S.*Typhimurium
  - Statistical analysis

SUPPLEMENTAL INFORMATION

Supplemental Information can be found online at https://doi.org/10.1016/j.cub.2020.05.083.

ACKNOWLEDGMENTS

This work was supported by the MRC (U105170648 to F.R., MC_UP_1201/8 to W.K.) and the Wellcome Trust (WT104752MA). We thank the LMB EM and light microscopy facilities for support in data acquisition and Ana Crespillo Casado for drawing the graphical abstract.

AUTHORS CONTRIBUTIONS

Experiments were performed and analyzed by C.J.E. and K.B.B. with CLEM performed and analyzed by W.K. C.J.E., S.M., and F.R. designed the study. C.J.E. and F.R. wrote the manuscript.

DECLARATION OF INTERESTS

The authors declare no competing interests.

Received: November 13, 2019
Revised: April 11, 2020
Accepted: May 27, 2020
Published: July 9, 2020

REFERENCES

1. Schaible, U.E., and Haas, A. (2019). Intracellular Niches of Microbes: A Microbes Guide Through the Host Cell, First Edition (Wiley).
2. Randow, F., MacMicking, J.D., and James, L.C. (2013). Cellular self-defense: how cell-autonomous immunity protects against pathogens. Science *340*, 701–706.
3. Gong, T., Liu, L., Jiang, W., and Zhou, R. (2020). DAMP-sensing receptors in sterile inflammation and inflammatory diseases. Nat. Rev. Immunol. *20*, 95–112.
4. Abdullah, Z., and Knolle, P.A. (2014). Scaling of immune responses against intracellular bacterial infection. EMBO J. *33*, 2283–2294.
5. Pizarro-Cerdá, J., Kühbacher, A., and Cossart, P. (2012). Entry of Listeria monocytogenes in mammalian epithelial cells: an updated view. Cold Spring Harb. Perspect. Med. *2*, a010009.
6. Fredlund, J., and Enninga, J. (2014). Cytoplasmic access by intracellular bacterial pathogens. Trends Microbiol. *22*, 128–137.
7. Mellouk, N., Weiner, A., Aulner, N., Schmitt, C., Elbaum, M., Shorte, S.L., Danckaert, A., and Enninga, J. (2014). Shigella subverts the host recycling compartment to rupture its vacuole. Cell Host Microbe *16*, 517–530.
8. Du, J., Reeves, A.Z., Klein, J.A., Twedt, D.J., Knodler, L.A., and Lesser, C.F. (2016). The type III secretion system apparatus determines the intracellular niche of bacterial pathogens. Proc. Natl. Acad. Sci. USA *113*, 4794–4799.
9. Birmingham, C.L., Smith, A.C., Bakowski, M.A., Yoshimori, T., and Brumell, J.H. (2006). Autophagy controls Salmonella infection in response to damage to the Salmonella-containing vacuole. J. Biol. Chem. *281*, 11374–11383.
10. Sekizawa, Y., Kubo, T., Kobayashi, H., Nakajima, T., and Natori, S. (1997). Molecular cloning of cDNA for lysenin, a novel protein in the earthworm Eisenia foetida that causes contraction of rat vascular smooth muscle. Gene *191*, 97–102.
11. Yamaji, A., Sekizawa, Y., Emoto, K., Sakuraba, H., Inoue, K., Kobayashi, H., and Umeda, M. (1998). Lysenin, a novel sphingomyelin-specific binding protein. J. Biol. Chem. *273*, 5300–5306.
12. Paz, I., Sachse, M., Dupont, N., Mounier, J., Cederfur, C., Enninga, J., Leffler, H., Poirier, F., Prevost, M.-C., Lafont, F., and Sansonetti, P. (2010). Galectin-3, a marker for vacuole lysis by invasive pathogens. Cell. Microbiol. *12*, 530–544.
13. Dupont, N., Lacas-Gervais, S., Bertout, J., Paz, I., Freche, B., Van Nhieu, G.T., van der Goot, F.G., Sansonetti, P.J., and Lafont, F. (2009). Shigella phagocytic vacuolar membrane remnants participate in the cellular response to pathogen invasion and are regulated by autophagy. Cell Host Microbe *6*, 137–149.
14. Thurston, T.L.M., Wandel, M.P., von Muhlinen, N., Foeglein, A., and Randow, F. (2012). Galectin 8 targets damaged vesicles for autophagy to defend cells against bacterial invasion. Nature *482*, 414–418.
15. Thurston, T.L., Boyle, K.B., Allen, M., Ravenhill, B.J., Karpiyevich, M., Bloor, S., Kaul, A., Noad, J., Foeglein, A., Matthews, S.A., et al. (2016). Recruitment of TBK1 to cytosol-invading Salmonella induces WIPI2-dependent antibacterial autophagy. EMBO J. *35*, 1779–1792.
16. Thurston, T.L.M., Ryzhakov, G., Bloor, S., von Muhlinen, N., and Randow, F. (2009). The TBK1 adaptor and autophagy receptor NDP52 restricts the proliferation of ubiquitin-coated bacteria. Nat. Immunol. *10*, 1215–1221.

CellPress
OPEN ACCESS

17. von Muhlinen, N., Akutsu, M., Ravenhill, B.J., Foeglein, Á., Bloor, S., Rutherford, T.J., Freund, S.M.V., Komander, D., and Randow, F. (2012). LC3C, bound selectively by a noncanonical LIR motif in NDP52, is required for antibacterial autophagy. Mol. Cell *48*, 329–342.
18. Bretscher, M.S. (1973). Membrane structure: some general principles. Science *181*, 622–629.
19. Segawa, K., and Nagata, S. (2015). An Apoptotic 'Eat Me' Signal: Phosphatidylserine Exposure. Trends Cell Biol. *25*, 639–650.
20. De Colibus, L., Sonnen, A.F.P., Morris, K.J., Siebert, C.A., Abrusci, P., Plitzko, J., Hodnik, V., Leippe, M., Volpi, E., Anderluh, G., and Gilbert, R.J. (2012). Structures of lysenin reveal a shared evolutionary origin for pore-forming proteins and its mode of sphingomyelin recognition. Structure *20*, 1498–1507.
21. Bokori-Brown, M., Martin, T.G., Naylor, C.E., Basak, A.K., Titball, R.W., and Savva, C.G. (2016). Cryo-EM structure of lysenin pore elucidates membrane insertion by an aerolysin family protein. Nat. Commun. *7*, 97–98.
22. Podobnik, M., Savory, P., Rojko, N., Kisovec, M., Wood, N., Hambley, R., Pugh, J., Wallace, E.J., McNeill, L., Bruce, M., et al. (2016). Crystal structure of an invertebrate cytolysin pore reveals unique properties and mechanism of assembly. Nat. Commun. *7*, 11598.
23. Kiyokawa, E., Baba, T., Otsuka, N., Makino, A., Ohno, S., and Kobayashi, T. (2005). Spatial and functional heterogeneity of sphingolipid-rich membrane domains. J. Biol. Chem. *280*, 24072–24084.
24. Kwiatkowska, K., Hordejuk, R., Szymczyk, P., Kulma, M., Abdel-Shakor, A.-B., Płucienniczak, A., Dołowy, K., Szewczyk, A., and Sobota, A. (2007). Lysenin-His, a sphingomyelin-recognizing toxin, requires tryptophan 20 for cation-selective channel assembly but not for membrane binding. Mol. Membr. Biol. *24*, 121–134.
25. Kulma, M., Hereć, M., Grudziński, W., Anderluh, G., Gruszecki, W.I., Kwiatkowska, K., and Sobota, A. (2010). Sphingomyelin-rich domains are sites of lysenin oligomerization: Implications for raft studies. Biomembranes *1798*, 471–481.
26. Xu, Y., Zhou, P., Cheng, S., Lu, Q., Nowak, K., Hopp, A.-K., Li, L., Shi, X., Zhou, Z., Gao, W., et al. (2019). A Bacterial Effector Reveals the V-ATPase-ATG16L1 Axis that Initiates Xenophagy. Cell *178*, 552–566.e20.
27. Lau, N., Haeberle, A.L., O'Keeffe, B.J., Latomanski, E.A., Celli, J., Newton, H.J., and Knodler, L.A. (2019). SopF, a phosphoinositide binding effector, promotes the stability of the nascent Salmonella-containing vacuole. PLoS Pathog. *15*, e1007959.
28. Marchesini, N., Luberto, C., and Hannun, Y.A. (2003). Biochemical properties of mammalian neutral sphingomyelinase 2 and its role in sphingolipid metabolism. J. Biol. Chem. *278*, 13775–13783.
29. Hofmann, K., Tomiuk, S., Wolff, G., and Stoffel, W. (2000). Cloning and characterization of the mammalian brain-specific, Mg2+-dependent neutral sphingomyelinase. Proc. Natl. Acad. Sci. USA *97*, 5895–5900.
30. Radtke, A.L., Delbridge, L.M., Balachandran, S., Barber, G.N., and O'Riordan, M.X.D. (2007). TBK1 protects vacuolar integrity during intracellular bacterial infection. PLoS Pathog. *3*, e29.
31. Isberg, R.R., Voorhis, D.L., and Falkow, S. (1987). Identification of invasin: a protein that allows enteric bacteria to penetrate cultured mammalian cells. Cell *50*, 769–778.
32. Isberg, R.R., and Falkow, S. (1985). A single genetic locus encoded by Yersinia pseudotuberculosis permits invasion of cultured animal cells by Escherichia coli K-12. Nature *317*, 262–264.
33. Kukulski, W., Schorb, M., Welsch, S., Picco, A., Kaksonen, M., and Briggs, J.A.G. (2011). Correlated fluorescence and 3D electron microscopy with high sensitivity and spatial precision. J. Cell Biol. *192*, 111–119.
34. Beuzón, C.R., Méresse, S., Unsworth, K.E., Ruíz-Albert, J., Garvis, S., Waterman, S.R., Ryder, T.A., Boucrot, E., and Holden, D.W. (2000). Salmonella maintains the integrity of its intracellular vacuole through the action of SifA. EMBO J. *19*, 3235–3249.
35. Park, D., Lara-Tejero, M., Waxham, M.N., Li, W., Hu, B., Galán, J.E., and Liu, J. (2018). Visualization of the type III secretion mediated *Salmonella*-host cell interface using cryo-electron tomography. eLife *7*, e39514.
36. Andrews, N.W., and Corrotte, M. (2018). Plasma membrane repair. Curr. Biol. *28*, R392–R397.
37. Jia, J., Abudu, Y.P., Claude-Taupin, A., Gu, Y., Kumar, S., Choi, S.W., Peters, R., Mudd, M.H., Allers, L., Salemi, M., et al. (2018). Galectins Control mTOR in Response to Endomembrane Damage. Mol. Cell *70*, 120–135.
38. Chu, C.T., Ji, J., Dagda, R.K., Jiang, J.F., Tyurina, Y.Y., Kapralov, A.A., Tyurin, V.A., Yanamala, N., Shrivastava, I.H., Mohammadyani, D., et al. (2013). Cardiolipin externalization to the outer mitochondrial membrane acts as an elimination signal for mitophagy in neuronal cells. Nat. Cell Biol. *15*, 1197–1205.
39. Mastronarde, D.N. (2005). Automated electron microscope tomography using robust prediction of specimen movements. J. Struct. Biol. *152*, 36–51.
40. Kremer, J.R., Mastronarde, D.N., and McIntosh, J.R. (1996). Computer visualization of three-dimensional image data using IMOD. J. Struct. Biol. *116*, 71–76.
41. Randow, F., and Sale, J.E. (2006). Retroviral transduction of DT40. Subcell. Biochem. *40*, 383–386.
42. Wandel, M.P., Pathe, C., Werner, E.I., Ellison, C.J., Boyle, K.B., von der Malsburg, A., Rohde, J., and Randow, F. (2017). GBPs Inhibit Motility of Shigella flexneri but Are Targeted for Degradation by the Bacterial Ubiquitin Ligase IpaH9.8. Cell Host Microbe *22*, 507–518.
43. Ader, N.R., and Kukulski, W. (2017). triCLEM: Combining high-precision, room temperature CLEM with cryo-fluorescence microscopy to identify very rare events. Methods Cell Biol. *140*, 303–320.
44. Ader, N.R., Hoffmann, P.C., Ganeva, I., Borgeaud, A.C., Wang, C., Youle, R.J., and Kukulski, W. (2019). Molecular and topological reorganizations in mitochondrial architecture interplay during Bax-mediated steps of apoptosis. eLife *8*, e40712.
45. Hohmann-Marriott, M.F., Sousa, A.A., Azari, A.A., Glushakova, S., Zhang, G., Zimmerberg, J., and Leapman, R.D. (2009). Nanoscale 3D cellular imaging by axial scanning transmission electron tomography. Nat. Methods *6*, 729–731.
46. Mastronarde, D.N., and Held, S.R. (2017). Automated tilt series alignment and tomographic reconstruction in IMOD. J. Struct. Biol. *197*, 102–113.

STAR★METHODS

KEY RESOURCES TABLE

REAGENT or RESOURCE	SOURCE	IDENTIFIER
Antibodies		
Goat polyclonal anti-galectin 8	R and D Systems	Cat# AF1305; RRID:AB_2137229
Mouse polyclonal anti-NDP52	Abnova	Cat# H00010241-B01P; RRID:AB_1571984
AlexaFluor-conjugated anti-goat or anti-mouse	Invitrogen	Various
Bacterial and Virus Strains		
Salmonella Typhimurium 12023	Gift from David Holden - Imperial College, London	N/A
Salmonella Typhimurium 12023 Δ*prgH*+pRI203	Gift from David Holden - Imperial College, London	N/A
Shigella flexneri Strain M90T	Gift from Chris Tang – Sir William Dunn School of Pathology, Oxford	N/A
Listeria monocytogenes Strain EGD, Bug600	Gift from Pascal Cossart, Institut Pasteur, Paris	N/A
Streptococcus pyogenes Strain H239	Gift from Imperial College, London	N/A
Enteropathogenic *E.coli* (EPEC) Strain E2348/69	Gift from David Holden – Imperial College, London	N/A
Chemically competent *E.coli* MC1061	Lab stock	N/A
Chemically competent *E.coli* BL21	Lab stock	N/A
Chemicals, Peptides, and Recombinant Proteins		
Lysenin(CTD)-GFP-His_6	This paper	N/A
Lysenin(CTD)-GFP-His_6	This paper	N/A
S.aureus bSMase (a.a.35-330)	This paper	N/A
Kanamycin	Merck	Cat# 420311
Gentamycin	Thermo Fisher Scientific	Cat #15750045
Isopropyl β-D-1-thioglalctopyranoside (IPTG)	SIGMA	Cat# I5502
Chicken Egg Sphingomyelin	Avanti Polar Lipids Inc.	Cat# 860061
Porcine Brain Phosphatidylcholine	Avanti Polar Lipids Inc.	Cat# 840053
Cholesterol	SIGMA	Cat# C8503
Optiprep	SIGMA	Cat# D1556
Sucrose	SIGMA	Cat# S0389
Polyethylenimine (PEI)	Polysciences	Cat# 23966-2
Complete Protease Inhibitor Cocktail	Roche	Cat #4693116001
Poly(ethyleneglycol) 1000 (PEG)	SIGMA	Cat# 81188
Glycyl-L-phenylalanine 2-naphthylamide (GPN)	SIGMA	Cat# G9512
L-leucyl-L-leucine methyl ester (LLOMe)	Cayman Chemicals	Cat# 16008
Listeriolysin O	Generon Ltd.	Cat# Pro-320
Saponin	Thermo Fisher Scientific	Cat# AC419231000
VECTASHIELD HardSet Antifade Mounting Medium with DAPI	Vector laboratories	Cat# H-1500
DRAQ5	eBioscience	Cat# 65-0880-92
ProLong gold antifade mountant	Invitrogen	Cat# P36930
Leibovitz's L-15 medium	GIBCO	Cat# 21083027
Lowicryl HM20 embedding kit	Polysciences, Inc.	Cat# 15924
Critical Commercial Assays		
Silver stain kit	BioRad	Cat# 161-0443

(*Continued on next page*)

CellPress
OPEN ACCESS

Continued

REAGENT or RESOURCE	SOURCE	IDENTIFIER
Deposited Data		
N/A		
Experimental Models: Cell Lines		
HeLa	European Collection of Authenticated Cell Cultures	RRID:CVCL_0030
THP-1	European Collection of Authenticated Cell Cultures	RRID:CVCL_0006
Murine embryonic fibroblasts	Gift from Chihiro Sasakawa, Univeristy of Tokyo	N/A
Oligonucleotides		
Primers used in this study are listed in Table S1		N/A
Recombinant DNA		
Plasmid: pETM-11 His_6-GFP	This study	N/A
Plasmid: pETM-11 His_6-GFP-Galectin 8	This study	N/A
Plasmid: pOPIN K $Lysenin^{CTD}$-GFP-His_6	This study	N/A
Plasmid: pOPIN K $Lysenin^{CTD}$K185A -GFP-His_6	This study	N/A
Plasmid: pOPIN B *S.aureus* bSMase aa 35-330	This study	N/A
Plasmid: M6P-GFP-$Lysenin^{CTD}$	This study	N/A
Plasmid: M6P-GFP-$Lysenin^{W20A}$	This study	N/A
Plasmid: M6P-GFP-$Lysenin^{W20A}$ K185A	This study	N/A
Plasmid: M6P-mCh-$Lysenin^{W20A}$	This study	N/A
Plasmid: M6P-mCh-Galectin-8	Gift from Michal Wandel – MRC LMB, Cambridge	N/A
Plasmid: M6P-YFP-Galectin-8	Wandel M.P et al., 2017	MW319
Plasmid: M6P-CFP-Galectin-8	Gift from Michal Wandel – MRC LMB, Cambridge	N/A
Plasmid: M6P-mCh-nSMase2	This study	N/A
Synthesized gene: *Eisenia fetida* Lysenin. Codon optimized for expression in human cells.	Life Technologies	N/A
Synthesized gene: *S.aureus* bSMase . Codon optimized for expression in *E.coli.*	Life Technologies	N/A
Software and Algorithms		
GraphPad Prism	https://www.graphpad.com/scientific-software/prism/	N/A
Zeiss ZEN	https://www.zeiss.com/microscopy/int/products/microscope-software/zen-lite.html	N/A
NIS Elements 4.40	https://www.microscope.healthcare.nikon.com/products/software/nis-elements	N/A
Imaris version 8	https://imaris.oxinst.com	N/A
FlowJo version 7	https://www.flowjo.com	N/A
aCOLyte3	http://www.synbiosis.com/acolyte-software/	N/A
Fiji	https://imagej.net/Fiji	N/A
SerialEM	https://bio3d.colorado.edu/SerialEM/ [39]	N/A
IMOD	https://bio3d.colorado.edu/imod/ [40]	N/A
Amira	Thermo Scientific	N/A
Other		
Hi-Trap Nickel column	GE Healthcare	Cat# 17524801
Superdex 200 16/600 column	GE Healthcare	Cat# 28989335
Superdex 75 16/600 column	GE Healthcare	Cat# 28989333

(*Continued on next page*)

Continued

REAGENT or RESOURCE	SOURCE	IDENTIFIER
Resource Q anion exchange column	GE Healthcare	Cat# 17117901
Vivaspin concentrators (10kDa cutoff)	Vivaproducts	Cat# VS2001
Mini-extruder	Avanti Polar Lipids Inc.	Cat# 610023
Nucleopore track etched membranes 400um and 100um	Whatman	Cat# WHA800282; Cat# WHA800309
Sapphire discs, 3mm	Engineering Office M.Wohlwend	Art. 405
Copper gold-plated support ring, 6 mm	Leica Microsystems	Cat# 16770111139
Nickel spacer ring, 3 mm, 2 mm hole	Leica Microsystems	Cat# 16770131268
Cover ring, 6 mm	Leica Microsystems	Cat# 16770111138
Protein A-coated 15 nm gold beads	Electron Microscopy Sciences	Cat# 25287
Copper EM grids with carbon film, 200 mesh	Agar Scientific	Cat# AGS160

RESOURCE AVAILABILITY

Lead Contact

Further information and requests for resources and reagents should be directed to, and will be fulfilled by, the Lead Contact Felix Randow (randow@mrc-lmb.cam.ac.uk).

Materials Availability

All unique reagents generated in this study are available from the Lead Contact with a completed Materials Transfer Agreement.

Data and Code Availability

All data generated and analyzed in this study are included in this published article and the associated supplementary information files.

EXPERIMENTAL MODEL AND SUBJECT DETAILS

Cell lines

Cells were grown in Iscove's Modified Dulbecco's Medium (IMDM) with 10% Fetal Calf Serum and Gentamicin (30 μg/ml). HeLa, THP-1 and MEF cells were grown in a static incubator at 37°C, 5% CO_2. THP1 cell differentiation was achieved by addition of PMA (phorbol 12-myristate 13-acetate) at 20 ng/ml for 72 hours. All stable cell lines were generated by retroviral transduction. All cell lines were tested to be mycoplasma free.

Bacteria

*S.*Typhimurium strains 12023 and 12023 Δ*prgH* + *Inv (encoded on* pRI203, gifts from David Holden, Imperial College, London) were grown at 37°C on LB agar plates or in Luria Broth (LB). *S.*Typhimurium strain 12023 Δ*prgH* + pRI203 lacks a functional SPI1-T3SS and expresses the invasin (*inv*) gene of *Yersinia pseudotuberculosis* [31, 32]. This strain is referred to as Δ*prgH* + *Inv* throughout this publication. *S.*Typhimurium 12023 strains either not expressing a fluorescent protein or expressing mCherry fluorescent protein or BFP from a pFPV25.1 plasmid were used. Strains harboring plasmids were grown in LB with 100 μg/ml ampicillin.

S.flexneri strain M90T (gift from Chris Tang, Sir William Dunn School of Pathology, Oxford) was grown at 37°C on TSB agar plates containing 0.003% congo red or in Tryptic Soy Broth (TSB). *S.flexneri* M90T either not expressing a fluorescent protein or expressing GFP from a pFPV25.1 plasmid were used. Strains harboring pFPV25.1 were grown in TSB with 100 μg/ml ampicillin.

L. monocytogenes strain EGD, BUG 600 (gift from Pascal Cossart, Institut Pasteur) was grown at 30°C on Brain Heart Infusion (BHI) agar plates or in BHI broth.

S.pyogenes strain H293 (gift from Imperial College, London) was grown at 37°C on blood agar plates or in Todd Hewitt Broth + 0.5% yeast.

EPEC strain E2348/69 (gift from David Holden, Imperial College, London) was grown at 37°C on LB agar plates or in LB.

E. coli strains MC1061 and BL21 were grown at 37°C on Tryptic Yeast Extract agar plates or in LB.

METHOD DETAILS

Plasmids

M6P plasmids were used to generate recombinant MLV for expression of proteins in mammalian cells [41]. Open reading frames encoding *Eisenia fetida* Lysenin or *Staphylococcus aureus* bacterial sphingomyelinase (aa. 35 – 330) were amplified by PCR from

synthesized genes, codon optimized for expression in human cells or expression in *E.coli*, respectively (Life Technologies). Mutations were generated by PCR. The open reading frame encoding neutral sphingomyelinase 2 was amplified by PCR from a human brain cDNA library. Open reading frames encoding GFP and Galectin-8 were amplified by PCR from plasmids, respectively [41, 42]. All plasmids were verified by sequencing. pOPIN B, pOPIN K and pETM-11 vectors were used for protein expression.

Sterile damage assays

For endosomal lysis, medium on cells was replaced with hypertonic medium (0.5 M sucrose in PBS, with 10% (w/v) polyethylene-glycol (PEG)) for 10 minutes at 37°C. Cells were then washed and incubated in 60% PBS for 3 minutes followed by incubation in complete IMDM medium for 20 minutes at 37°C. For lysosomal lysis, medium on cells was replaced with 333 μM Glycyl-L-phenylalanine 2-naphthylamide (GPN) for 10 minutes at 37°C.

For treatment of cells with L-leucyl-L-leucine methyl ester (LLOMe), medium on cells was replaced with medium containing 250 μM LLOMe (Cayman Chemicals) and incubated at 37°C for 15 minutes, washed once with PBS and fixed.

For treatment of cells with Listeriolysin O, cells were washed twice with ice-cold medium and incubated with medium containing 300 ng/ml Listeriolysin O (Generon Ltd) for 45 minutes on ice. Cells were placed at 37°C for 20 minutes, washed once with PBS and fixed.

Following treatments, cells were fixed in 4% paraformaldehyde at 22°C for 15 minutes, washed twice and quenched in 100 mM glycine in PBS.

Bacterial infections

*S.*Typhimurium strains 12023 and 12023 Δ*prgH* + *Inv* were grown overnight at 37°C, 180 rpm in Luria Broth (LB) with the addition of relevant antibiotics where appropriate (100 μg/ml ampicillin for 12023 Δ*prgH* + *Inv* strain and for strains harboring a fluorescent protein expression plasmid). 3.5 hours prior to infection, sub-inoculation was carried out at a ratio of 1:33 into fresh LB. HeLa cells, MEF cells and mature THP-1 cells in 24-well plates were infected with 20 μL of sub-culture per well for 15 minutes. Cells were washed twice in phosphate buffered saline (PBS, pH 7.4) and cultured in IMDM containing 100 μg/ml gentamycin.

S.flexneri strain M90T was grown overnight at 37°C, 180 rpm in Tryptic Soy Broth (TSB) with the addition of relevant antibiotics where appropriate (100 μg/ml ampicillin for strains harboring GFP expression plasmid). 2.5 hours prior to infection, bacteria were sub-cultured at a ratio of 1:100 into fresh TSB. HeLa cells grown in 24-well plates were infected with 100 μL of sub-culture and centrifuged for 10 minutes at 2,000 rpm, 20°C followed by incubation at 37°C for 30 minutes. Cells were washed twice in PBS and placed into IMDM containing 100 μg/ml gentamycin.

L. monocytogenes Strain EGD, BUG 600 was grown overnight in Brain Heart Medium at 30°C, 180 rpm. Cultures were then washed in PBS and resuspended in antibiotic-free IMDM medium immediately before 10 μL of culture was used to infect HeLa cells. Samples were centrifuged for 10 minutes at 2,000 rpm and incubated at 37°C for 60 minutes. Cells were washed twice in PBS and placed into IMDM containing 100 μg/ml gentamycin.

S.pyogenes, strain H293 was grown overnight in 5 mL Todd Hewitt Broth (SIGMA) + 0.5% yeast at 37°C, without shaking. Cultures were then washed in PBS and resuspended in an equivalent volume of antibiotic-free IMDM medium immediately before 20 μL of culture was used to infect HeLa cells at 37°C for 1 hour. Cells were then washed twice in PBS and placed into IMDM containing 100 μg/ml gentamycin.

EPEC strain E2348/69 was grown overnight in LB at 37°C, 180 rpm. 3.5 hours prior to infection, sub-inoculation at 1:33 was carried out into fresh LB. HeLa cells and PMA-differentiated THP-1 cells in 24-well plates were infected with 40 μL of sub-inoculation per well and centrifuged at 2,000 rpm for 5 minutes, 20°C. Medium remained unchanged throughout the infection.

Where appropriate, cells were fixed at relevant time points post infection as described for sterile damage assays.

Enumeration of intracellular *S.* Typhimurium (colony forming unit assay)

At relevant time points post infection with *S.*Typhimurium, HeLa cells seeded in triplicate wells were lysed in 1 mL cold PBS containing 0.1% Triton X-100. Serial dilutions were plated on TYE agar plates. Plates were incubated overnight at 37°C and colonies were counted using an aCOLyte3 colony counter (Synbiosis).

Antibody staining

Cells were seeded on glass coverslips prior to infection or sterile damage treatment. Following fixation (as described in sterile damage assays), cells were permeabilised and blocked in PBS containing 0.1% (w/v) Saponin and 2% (w/v) BSA for 1 hour. Coverslips were then incubated in primary antibody diluted in PBS containing 0.1% (w/v) Saponin and 2% (w/v) BSA followed by an Alexa-conjugated secondary antibody for 1 hour. Coverslips were then mounted either in DAPI mounting medium (Vector laboratories) or ProLong Gold Antifade Mountant (Invitrogen) for confocal imaging or super resolution microscopy, respectively.

Microscopy

Confocal images were taken with a 63X, 1.4 numerical aperture (NA) oil objective on a Zeiss 780 microscope. Super resolution images were taken using an Elyra S1 structured illumination microscope (Carl Zeiss Microscopy Ltd., Cambridge, UK). SIM Images were obtained using a 63X, 1.4 NA oil objective with grating projections at 3 rotations and 5 phases in accordance with the manufacturer's instructions. Super resolution images were calculated using Zeiss ZEN software. Live-cell imaging was achieved using a 60X, water

objective of Nikon Eclipse Ti equipped with an Andor Revolution XD system and a Yokogawa CSU-X1 spinning disk unit. Movies were analyzed using Imaris software version 8. Tracking individual bacteria was achieved using spot detection on this software. The mean fluorescence intensity of each fluorescence channel (488 nm and 561 nm) at each time point was normalized between 1- 100.

Live-cell super resolution imaging was achieved using a 100X super resolution Apo TIRF oil objective on a Nikon Eclipse Ti2 with a VisiTech iSIM high speed super resolution system. Movies were analyzed using NIS Elements 4.4.

Protein expression and purification

His_6-GFP and His_6-GFP-Galectin-8 (both in the pETM-11 vector), $Lysenin^{CTD}$-GFP-His_6 and $Lysenin^{CTD,K185A}$-GFP-His_6 (both in the pOPIN K vector) and *Staphylococcus aureus* bacterial SMase aa. 35 - 330 (in the pOPIN B vector) were expressed in BL21 *E.coli.* Cells were grown at 30°C in 2xTY supplemented with 30 μg/ml kanamycin. Cultures were induced with IPTG (400 μM) at 18°C and harvested after 16 hours. Proteins were purified by immobilised metal-affinity chromatography. His_6-GFP and His_6-GFP-Galectin-8 were further purified by size exclusion chromatography using a Superdex 200 16/600 column (GE Healthcare) in 20 mM Tris pH 7.4, 150 mM NaCl, 2 mM DTT. $Lysenin^{CTD}$-GFP-His_6 and $Lysenin^{CTD,K185A}$-GFP-His_6 were further purified by anion exchange chromatography using a Resource Q column (GE Healthcare) with 20 mM Tris pH 8.5, 2 mM DTT, 1 M NaCl buffer. *S.aureus* bacterial sphingomyelinase was further purified by size exclusion chromatography using a Superdex 75 column (GE Healthcare) in 20 mM MES, 4 mM DTT, 200 mM NaCl, 2 mM $MgCl_2$, 5% (V/V) glycerol, pH 6. $MgCl_2$ was added to the concentrated protein at a final concentration of 5 mM.

Surface binding assays

HeLa cells were seeded on coverslips and appropriate samples were pretreated with recombinant bacterial sphingomyelinase (bSMase) at 7 μg/ml in IMDM for 30 minutes at 37°C and washed with PBS. Coverslips were incubated with recombinant proteins (10 μg/ml) for 30 minutes at 4°C. Cells were washed twice with PBS, fixed in 4% PFA as for sterile damage assays, mounted and captured by confocal microscopy as described above.

Alternatively, HeLa cells in 24-well plates were detached with trypsin and appropriate samples were pretreated with bSMase (7 μg/ml in PBS) and washed twice in PBS. Samples were then incubated with recombinant proteins (10 μg/ml) for 30 minutes at 4°C, washed and fixed as above. Cells were analyzed on BD LSR Fortessa flow cytometer. Results were analyzed using FlowJo version 7.

Liposome flotation assays

Sphingomyelin from egg yolk, Phosphatidylcholine (PC) from porcine brain and Cholesterol (Chol) were obtained from Avanti Polar Lipids. Lipids were mixed in chloroform at the following ratios: PC and Chol (60:40); sphingomyelin, PC and Chol (50:10:40). Solvent was evaporated under nitrogen flow and lipids were further dried for 1 hour under a vacuum. Lipid mixtures were rehydrated in rehydration buffer (50 mM HEPES, 100 mM KoAC, 1 mM DTT, 10% OptiPrep (SIGMA)) added to a final lipid concentration of 1 mg/ml for 1 hour. Liposomes were prepared with a Mini-Extruder (Avanti Polar Lipids, Inc.) using Nucleopore track-etched membranes with 400nm followed by 100nm pores (Whatman). Liposomes were validated by dynamic light scattering using a DynaPro Plate Reader II instrument (Wyatt Technology). Liposomes containing 10% OptiPrep were incubated with recombinant proteins at 20 μg/ml for 1 hour at r.t.. OptiPrep was then added to a final concentration of 30% and the mixture was overlaid with a 10% OptiPrep layer and a 0% Optiprep layer (rehydration buffer only). Liposomes were floated in a SW60Ti swinging bucket rotor (51,000 rpm, 30 minutes, 4°C). Liposomes floating at the 10%–0% Optiprep interface were collected and washed by re-layering of the OptiPrep gradient and re-floating. Floating liposomes were collected and bound proteins were precipitated via methanol:chloroform extraction.

Correlative Fluorescence Microscopy and Electron Tomography

HeLa cells co-expressing mCherry-$Lysenin^{W20A}$ and YFP-Galectin-8 were seeded on carbon-coated 3 mm sapphire discs (Engineering Office M. Wohlwend). Cells were infected with *S.* Typhimurium as for microscopy assays. At approximately 30 – 40 minutes p.i., infected cells were vitrified by high-pressure freezing using a HPM100 (Leica Microsystems). For high-pressure freezing, sapphire discs with cells were assembled on a copper gold-plated support ring (Leica Microsystems), covered with a nickel spacer ring (Leica Microsystems), a second sapphire disc and a cover ring (Leica Microsystems), as described [43]. Samples were processed as described before [44]. In brief, freeze substitution was done using 0.008% uranyl acetate in acetone followed by embedding in Lowicryl HM20 (Polysciences) resin in an AFS2 (Leica Microsystems). 300 nm sections were cut using an Ultracut E microtome (Reichert) and a diamond knife (Diatome) and collected onto 200 mesh carbon-coated copper grids (Agar Scientific). Grids were imaged on a TE2000-E widefield microscope (Nikon) using a 100x TIRF objective (Nikon) on a Neo sCMOS camera (Andor). Fluorescence signals were imaged using a Niji LED light source (bluebox optics) and filter sets 49002 ET EGFP and 49005 ET DSRED (Chroma) for YFP and mCherry, respectively. Prior to EM, grids were incubated with protein A-coated 15 nm gold beads (EMS) and washed 3 times with distilled water. Dual-axis tomographic tilt series were acquired on a Tecnai F20 (FEI) electron microscope at 1.1 nm pixel size and 1 degree increment, approximately ± 60°, in bright field-STEM mode as described before [43, 45] using SerialEM [39]. Tomograms were reconstructed using IMOD [40, 46]. Segmentation models were generated by manual tracing, followed by extensive simplification and smoothening of the generated surfaces, in Amira (Thermo Scientific). Segmentation models are inverted in z relative to the original tomographic volumes.

CellPress
OPEN ACCESS

QUANTIFICATION AND STATISTICAL ANALYSIS

Quantification of bacteria

Three methods of quantification were used. Marker positive bacteria were scored by eye using a Zeiss Axioscope upright fluorescence microscope with a 100X, 1.3 NA oil objective; three independent experiments with three replicate coverslips were performed. Marker positive bacteria were scored automatically by a Nikon High Content microscope using a 20X, 0.75 NA air objective with NIS Elements 4.4 software; three independent experiments with three replicates were performed. Marker positive bacteria were also scored by eye from images captured by a Nikon High Content microscope using a 40X, 0.95 NA air objective; three independent experiments with three replicates were performed. Method of quantification is indicated in the Figure Legends. Graphs show mean ± SEM.

Enumeration of intracellular *S.*Typhimurium

To score invasion and replication of *S.*Typhimurium, cells from triplicate wells were lysed as described in the colony forming unit assay method and bacteria were plated in duplicate on TYE agar. Each experiment was performed three times. Bacterial colonies were counted using the aCOLyte3 colony counter (Synbiosis). Graphs show mean ± SEM for combined datasets.

Statistical analysis

All data were tested for statistical significance with Prism software (GraphPad Prism 7). Unless otherwise stated, all experiments were performed at least three times and the data were combined and presented as mean ± SEM. Statistical details, including sample size (n), are reported in the Figure Legends.

Current Biology, Volume *30*

Supplemental Information

Transbilayer Movement of Sphingomyelin Precedes Catastrophic Breakage of Enterobacteria-Containing Vacuoles

Cara J. Ellison, Wanda Kukulski, Keith B. Boyle, Sean Munro, and Felix Randow

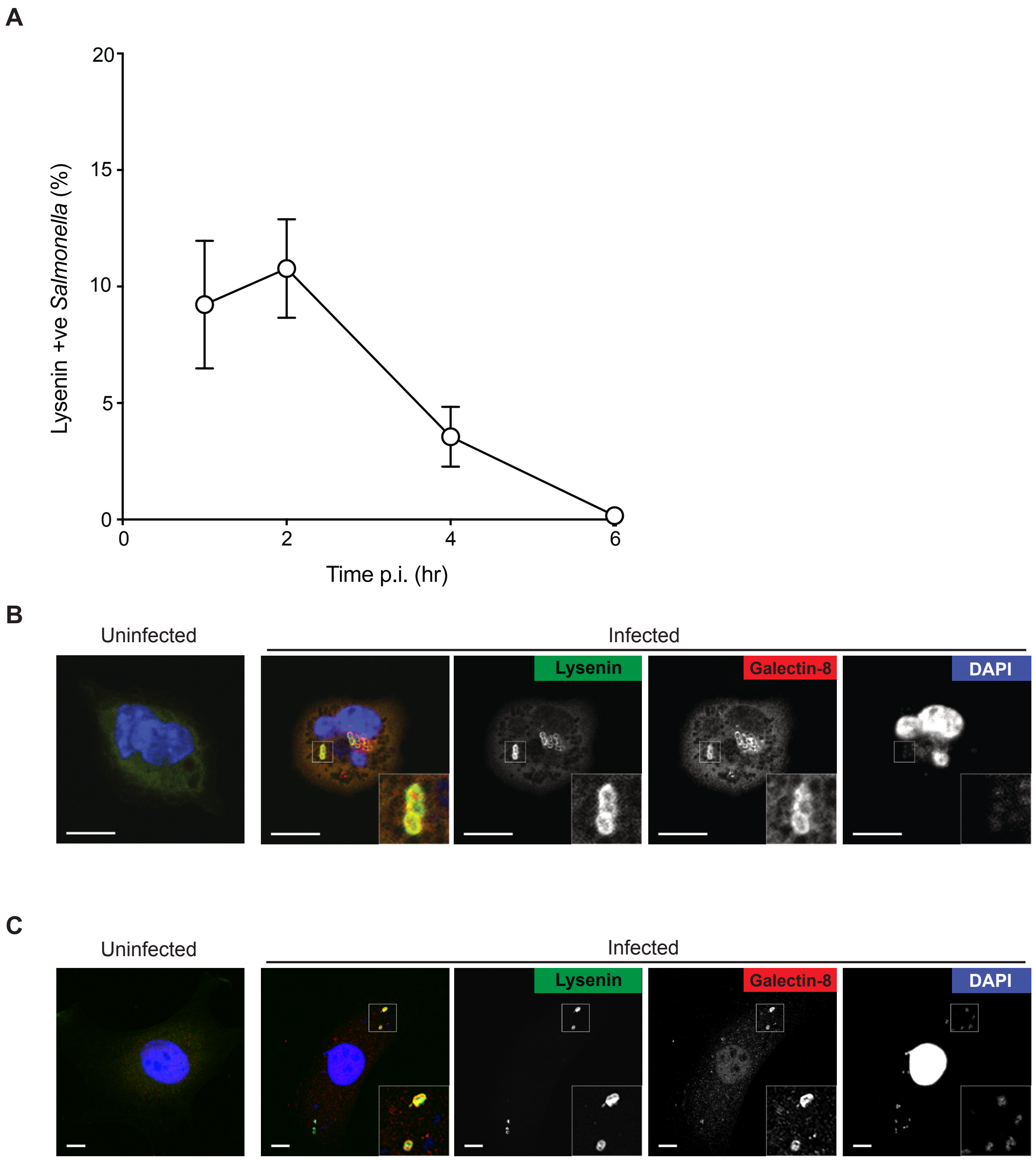

Figure S1: Lysenin recruitment to SCVs in HeLa, myeloid cells and fibroblasts, related to Figure 2

(A) Quantification of Lysenin^{W20A} recruitment to *S.* Typhimurium in HeLa cells at indicated time points p.i.. Mean ± SEM of triplicate coverslips from three independent experiments. Quantification by eye using widefield microscopy. n>100 bacteria counted per coverslip.

(B) Confocal micrographs of THP-1 cells expressing GFP-Lysenin^{W20A} infected with *S.* Typhimurium and stained for galectin-8 at 60 minutes p.i.. Scale bar, 10µm.

(C) Confocal micrographs of mouse embryonic fibroblast cells expressing GFP-Lysenin^{W20A} infected with *S.* Typhimurium and stained for galectin-8 at 60 minutes p.i.. Scale bar, 10µm.

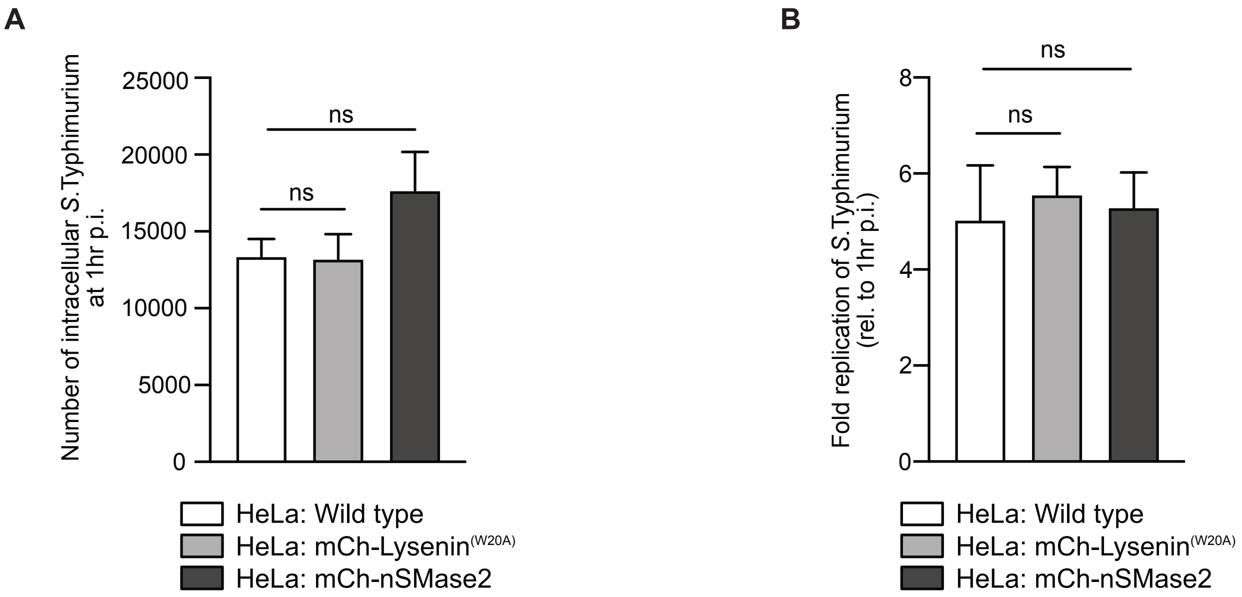

Figure S2: Ectopic expression of Lysenin or nSMase2 does not affect invasion by, or replication of, *S.* Typhimurium, related to Figure 2

(A) Quantification of intracellular *S*. Typhimurium in wild-type HeLa cells or HeLa cells expressing mCh-Lysenin^{W20A} or mCh-nSMase2 at 1 hour p.i.. Bacteria were counted in a colony forming unit assay. Mean ± SEM. One-way ANOVA with Dunnett's T3 multiple comparison test, ns = non-significant.

(B) Fold replication of *S*. Typhimurium in wild-type HeLa cells or HeLa cells expressing mCh-Lysenin^{W20A} or mCh-nSMase2 at 6hr p.i. relative to 1 hour p.i.. Bacteria were counted in a colony forming unit assay. Mean ± SD. One-way ANOVA with Dunnett's T3 multiple comparison test, ns = non-significant.

**Oligonucleotides**
Primer - Lysenin forward:<br>GGCCGGACATGTCCTCTGCCAAGGCCGCCGAGGGC
Primer - Lysenin reverse:<br>CCGGCCGCGGCCGCTCAGCCCACGACTTCCAGGAT
Primer - Lysenin CTD forward:<br>GGCCGGACATGTCCATCATCCTGGGAAAGACCGAG
Primer - nSMase2 forward:<br>GGCCGGACATGTCCGTTTTGTACACGACCCCCTTTCC
Primer - nSMase2 reverse:<br>CCGGCCGCGGCCGCCTATGCCTCCTCCTCCCCCGAAGA
Primer - Lysenin W20A mutation forward:<br>GATGTGGTGGCCGTG**GCG**AAAGAGGGCTATGTG
Primer - Lysenin W20A mutation reverse:<br>CACATAGCCCTCTTT**CGC**CACGGCCACCACATC
Primer - Lysenin K185A mutation forward:<br>ACCGTGGTGTCCAGA**GCG**AGCTGGCCTGCCGCC
Primer - Lysenin K185A mutation reverse:<br>GGCGGCAGGCCAGCT**CGC**TCTGGACACCACGGT
Primer - Lysenin CTD-GFP into pOPIN K at NcoI,HindIII sites - forward:<br>GGCCGGCCATGGCCATCATCCTGGGAAAGACC
Primer - Lysenin CTD-GFP into pOPIN K at NcoI,HindIII sites - reverse:<br>CCGGCCAAGCTTTCAATGATGATGATGATGATGGCCGGAGCCCTTGTACAGCTCGTCCAT
Primer - GFP into pET-M11 forward:<br>GGCCGGACATGTCCGTGAGCAAGGGCGAGGAGCTG
Primer - GFP into pET-M11 reverse:<br>CCGGCCGCGGCCGCTTACTTGTACAGCTCGTCCAT
Primer - GFP-Galectin 8 into pET-M11 forward:<br>GGCCGGACGCGTATGATGTTGTCCTTA
Primer - GFP-Galectin 8 into pET-M11 reverse:<br>CCGGCCGCGGCCGCCTACCAGCTCCTTACTTC
Primer - bSMase into pOPIN B forward:<br>*AAGTTCTGTTTCAGGGCCCG*ATGGAAAGCAAAAAAGATGACACCGAT
Primer - bSMase into pOPIN B reverse:<br>*ATGGTCTAGAAAGCTTTA*TTTGCTGTAGGCTTTAATC

Table S1: Oligonucleotides used in this study, related to STAR Methods.
